# SRPK2 Expression and Beta-Amyloid Accumulation Are Associated With BV2 Microglia Activation

**DOI:** 10.3389/fnint.2021.742377

**Published:** 2022-01-28

**Authors:** Ziqi Tian, Wenfang Zeng, Cuihuan Yan, Qiang Li, Nan Li, Lin Ruan, Jie Li, Xiaoguang Yao, Si Li

**Affiliations:** ^1^Hebei Key Laboratory of Integrative Medicine on Liver-Kidney Patterns, College of Integrative Medicine, Institute of Integrative Medicine, Hebei University of Chinese Medicine, Shijiazhuang, China; ^2^Department of Nephrology, The First Hospital of Hebei Medical University, Shijiazhuang, China; ^3^College of Acupuncture and Massage, Hebei University of Chinese Medicine, Shijiazhuang, China; ^4^Department of Technology, Hebei University of Chinese Medicine, Shijiazhuang, China

**Keywords:** Alzheimer's disease, SRPK2, microglia, neuroinflammation, Akt

## Abstract

**Introduction:**

The extracellular deposition of β-amyloid (Aβ) is a pathological hallmark in Alzheimer's disease (AD), which induces microglial activation in the pathology of AD. The expression of serine/threonine-protein kinase 2 (SRPK2) is increased in the brain tissues of patients with AD. In this study, we examined the effect of SRPK2 in the activation of microglia.

**Methods:**

Microglia (BV2) cells were cultured and the expression of SRPK2 was enhanced by transfection of SRPK2 recombinant vectors or knockdown by SRPK2 small interfering RNA (siRNA). The cells were stimulated by lipopolysaccharide (LPS) + interferon-γ (IFN-γ) or Aβ *in vitro*, generating inflammatory cytokines [tumor necrosis factor-α (TNF-α), interleukin (IL)-10, and IL-6], which were investigated by real-time quantitative PCR (qPCR) and ELISA. The proliferation ability of the BV2 cells with/without SRPK2 expression was evaluated by WST-1 under pressure in the presence of Aβ. The effects of SRPK2 on microglia polarization were evaluated by investigating the expression of CD16/32 and CD206 by western blot and the expression of ionized calcium-binding adapter molecule-1 (IBA-1) and arginase-1 (Arg-1) by immunofluorescence. Hippocampal cells HT-22 were cultured with a BV2 cell (with/without SRPK2 expression)-derived medium stimulated by Aβ or LPS + IFN-γ, prior to the evaluation of HT-22 cytotoxicity by assessment of cell viability. Possible relationships between Akt and SRPK2 in the BV2 cells were investigated by western blot.

**Results:**

The expression of SRPK2 was related to the phenotype polarization changes of microglia with increased expression of CD16/32 and IBA-1. The expression of proinflammatory cytokines IL-6 and TNF-α was increased, whereas the expression of anti-inflammatory cytokine IL-10 was decreased in the BV2 cells with SRPK2 overexpression. Moreover, with the expression enhancement of SRPK2, the BV2 cells had a higher proliferation rate. Aβ treatment can promote SRPK2 expression in BV2 cells. Aβ or LPS + IFN-γ promoted the production of cytokines IL-6 and TNF-α but decreased cytokine IL-10 in the BV2 cells. SRPK2 deficiency alleviated the cytotoxic effects of Aβ or LPS + IFN-γ exposed microglia on HT22 cells. In addition, the activated Akt pathway promoted the expression of SRPK2 in the BV2 cells.

**Conclusion:**

Our data have found that enhanced SRPK2 expression contributed to the proinflammatory activation of microglia. Thus, SRPK2 may be a key modulating pathway of inflammatory mediators in AD pathology.

## Introduction

Alzheimer's disease (AD), which is pathologically characterized by brain tissue atrophy, amyloid plaque formation [β-amyloid (Aβ)], neurofibrillary tangles (mainly tau protein) formation, neuronal synapse loss, etc., was first described by the German pathologist Alzheimer in 1906. The pathophysiology of AD has remained controversial; the effect of Aβ and tau protein cannot explain all the pathology of AD. In recent years, neuroinflammation has been identified to play an important role in the development of AD (Bagyinszky et al., 2017). The role of glial cell activation, especially microglia cells, in neuroinflammation has been widely confirmed (Kim et al., 2020). Microglia are the innate immune cells of the central nervous system (Ginhoux et al., 2010), acting as phagocytes and playing a key role in immune defense and injury response (Nayak et al., 2014). Moreover, microglia participate in the developmental regulation of neural circuits by engulfing and removing unwanted neurons and synapses (Frost and Schafer, 2016). The role of microglia has also been identified in multiple neurodegenerative diseases including AD pathogenesis (Maragakis and Rothstein, 2006; Ransohoff and Perry, 2009; Glass et al., 2010).

Generally, microglia can be transformed into activated states: the classically activated (M1) subtype contributes to inflammatory responses and the alternatively activated (M2) subtype plays anti-inflammatory roles. Previous studies have found that microglia may promote the pathological changes of AD through multiple pathways. Aβ peptides have been found to induce the activation of primary microglia, which can surround and degrade the plaques for Aβ by phagocytosis under the M2 activation phenotype to protect neurons. However, M2 microglia with Aβ phagocytic capabilities most likely shift microglia into the proinflammatory M1 state. The activated M1 state microglia inhibit the phagocytosis process and stimulate an inflammatory response with the production of proinflammatory cytokines such as interferon-γ (IFN-γ), interleukin-1β (IL-1β), and tumor necrosis factor-α (TNF-α), which eventually lead to neurotoxicity by inducing the death of neuronal cells and increasing amyloid deposits (Terwel et al., 2011). Furthermore, some proinflammatory cytokines secreted by microglia, such as IL-1β, interleukin-6 (IL-6), and TNF-α, can modify the phosphorylation pattern of tau protein not only to change its conformation but to change its structure and function, both of which accelerate the formation of tangles. Therefore, microglia may play a vital role in the pathological changes in AD. Although the molecular mechanism of microglia activation remains unknown, some studies have revealed that various kinases could be the key players in microglia activation in AD pathology (Lee and Suk, 2018). In view of this, Ofengeim et al. (2017) found that Receptor-interacting serine/threonine-protein kinase 1 (RIPK1) expression can activate the microglial response in AD, while Keaney et al. (2019) has suggested that tyrosine kinase can regulate microglia phagocytosis in AD.

Serine/threonine-protein kinase 2 (SRPK2) is a serine/threonine-protein kinase that can specifically phosphorylate substrates rich in serine-arginine residues. SRPK2 has been found to be widely expressed in the nervous system and the increased expression of SRPK2 has been identified in AD (Hong et al., 2012; Wang et al., 2017). In previous studies, we found the upregulated expression of SRPK2 in the brain tissue of an AD mice model, which contributed to the progression of AD pathology, with decreased cognitive ability (Yao et al., 2019). Therefore, we hypothesized that increased expression of SRPK2 in the brain could be related to the activation of microglia and participation in the progression of AD. In this study, we investigated the potential effects of SRPK2 in the pathology of microglia (flowchart of experiment design in Supplementary Figure 1). The results showed that Aβ can induce the expression of SRPK2, which promotes the activation of microglia, with polarization, proliferation, and production of inflammatory cytokines. Moreover, the expression of SRPK2 could be activated through the Akt pathway in microglia cells.

## Materials and Methods

### β-Amyloid Oligomer Preparation

β-amyloid was prepared as described in a previous study (Maezawa et al., 2011). Briefly, Aβ42 peptide (GL Biochem, Shanghai, China) was dissolved in 100% 1,1,1,3,3,3-hexafluoro-2-propanol (HFIP) at a concentration of 1 mM and then it was air-dried to remove HFIP at room temperature. The residues were dissolved in 20 μl dimethyl sulfoxide (DMSO) containing F12 medium to obtain a 100 μM stock solution. The solution was incubated at 4°C overnight and then centrifuged at 14,000 × g for 10 min at 4°C. The supernatant containing Aβ oligomers was collected, which was confirmed by immunoblot for further experiments (Jian et al., 2019).

### Microglia Cell Cultures and Treatment

A mouse microglia cell line (BV2) and a mouse neuron cell line (HT-22) were purchased from the China Center for Type Culture Collection (CCTCC) (Wuhan, China) and were maintained in Dulbecco's Modified Eagle's Medium (DMEM) (HyClone, Marlborough, Massachusetts, USA), supplemented with 10% ultracentrifuged fetal bovine serum (FBS) (Invitrogen, Carlsbad, California, USA), penicillin (Invitrogen, Carlsbad, California, USA), and streptomycin (Invitrogen, Carlsbad, California, USA) at 37°C in a humidified atmosphere of 5% carbon dioxide (CO_2_).

To induce the inflammatory response, the BV2 cells were incubated in a medium containing 100 ng/ml lipopolysaccharides (LPSs) (Beyotime Biotechnology, Shanghai, China) and 20 units/ml mouse IFN-γ (Beyotime Biotechnology, Shanghai, China) for 24 h (Culbert et al., 2006). The BV2 cells were treated with Aβ (10 μM) in a serum-free culture medium for 24 h (Jian et al., 2019). In addition, the BV2 cells were treated with Akt activator (SC79) R&D, Minneapolis, MN, USA at 5 μM (Sladitschek and Neveu, 2015).

### Neuronal Cell Treatment

The BV2 cells were seeded in a 96-well plate (5,000 cells/well) and were stimulated by Aβ or LPS + IFN-γ, respectively, for 24 h in a serum-free culture medium, while the controls were treated with DMSO (controls for Aβ) or phosphate-buffered saline (PBS) (controls for LPS + IFN-γ). After incubation, the culture medium of the BV2 cells stimulated by Aβ or LPS + IFN-γ (condition medium Aβ and condition medium LPS + IFN-γ, respectively) was collected and filtered with a sterile filter (0.22 μm) to remove cells and cell debris. To investigate the effect of BV2 on the neuronal cells, HT-22 cells were seeded in a 96-well plate at 5,000 cells per well and were treated with the condition culture medium (from Aβ or condition LPS- + IFN-γ-treated BV2 cells, respectively) and 50% fresh culture medium, as described above for 48 h.

### Cell Viability Assay

The cells were seeded in 96-well plates at 5,000 cells/well and received different treatments for 72 h. Thereafter, a Water Soluble Tetrazolium (WST-1) assay (Beyotime Biotechnology, Shanghai, China) was employed to evaluate cell viability in accordance with the manufacturer's instructions. Absorbance was measured at 450 nm using a multimode plate reader. The data were used to determine cell viability rates.

### Enzyme-Linked Immunosorbent Assay

The BV2 cells received various pretreatments including (a) SRPK2 expression enhancement by transfection of recombinant lentiviral vectors; (b) knockdown expression of SRPK2 by transfection of small interfering RNA (siRNA); (c) treatment with Aβ for 72 h; or (d) treatment with LPS + IFN-γ for 72 h. Thereafter, the culture medium was collected and the samples for cytokine levels, including IL-6, IL-10, and TNF-α, were prepared using the kit purchased from Beyotime Biotechnology (Shanghai, China). Briefly, 100 μl of samples or standards were added to an antibody-coated 96-well plate containing 100 μl of biotinylated detection antibody. The plate was then incubated at room temperature for 1 h. After incubation, the liquid was removed and the wells were washed with PBS before 100 μl streptavidin complex reagent was added to the wells and incubated at room temperature for 1 h. Next, 90 μl of HRP 3,3'5,5'-Tetramethylbenzidine (TMB) substrate solution was added to the wells and incubated at room temperature for 20 min. A stop solution of 50 μl was added to the wells and a multimode plate reader was used to evaluate the absorbance at 450 nm. The standard curves were generated based on the OD values of standard reagents and the cytokine levels were determined based on the standard curve.

### Reverse Transcription Quantitative-PCR (RT-qPCR)

Total RNA was extracted from cells or tissues using a Trizol method (Beyotime Biotechnology, Shanghai, China) following the manufacturer's instructions and was reversely transcribed into complementary DNA (cDNA) using the SuperScript™ III Reverse Transcriptase (Invitrogen; Thermo Fisher Scientific; Waltham, MA, USA). A qPCR (SYBR™ Green Master Mix; Thermo Fisher Scientific; cat. no. 4309155) was subsequently performed using the ABI PRISM 7500 Real-Time PCR System (Applied Biosystems; Thermo Fisher Scientific; Waltham, MA, USA), according to the manufacturer's protocol. The following primer pairs were used for the qPCR: Glyceraldehyde 3-phosphate dehydrogenase (GAPDH) forward: 5′-AATGGATTTGGACGCATTGGT-3′ and reverse: 5′-TTTGCACTGGTACGTGTTGAT-3′; IL-10 forward: 5′-GCTCTTACTGACTGGCATGAG-3′ and reverse: 5′-CGCAGCTCTAGGAGCATGTG-3′; TNF-α forward: 5′-GGTGCCTATGTCTCAGCCTCTT-3′ and reverse: 5′-GCCATAGAACTGATGAGAGGGAG-3′; and IL-6 forward: 5′-TAGTCCTTCCTACCCCAATTTCC-3′ and reverse: 5′-TTGGTCCTTAGCCACTCCTTC-3′.

The gene expression levels were quantified using the 2^−ΔΔCq^ method and normalized to the internal reference gene *GAPDH*. Each experiment was performed in triplicate.

### Cell Cycle Assay

The BV2 cells received various pretreatments including (a) SRPK2 expression enhancement by transfection of recombinant lentiviral vectors; (b) knockdown expression of SRPK2 by transfection of siRNA; (c) treatment with Aβ for 72 h; or (d) treatment with LPS + IFN-γ for 72 h. Thereafter, the cells were harvested. To perform the cell cycle assay, the cells were fixed in cold 70% ethanol for 30 min at 4°C and were washed and suspended in 500 μl PBS buffer. Thereafter, 50 μl RNase (100 μg/ml) and 200 μl Propidium iodide (PI) (50 μg/ml) were added to the cell suspensions. Finally, the cell cycles were evaluated using the FACScan Flow Cytometer (Becton Dickinson, Franklin Lakes, New Jersey, USA) in accordance with the instructions.

### Western Blotting

To investigate the effect of SRPK2 expression on activation of microglial cells, the BV2 cells received pretreatments including (a) SRPK2 expression enhancement by transfection of recombinant lentiviral vectors and (b) knockdown expression of SRPK2 by transfection of siRNA. To investigate the relationship between SRPK2 and Akt, the BV2 cells received various pretreatments including (a) knockdown expression of Akt by transfection of siRNA; (b) treatment with Aβ for 24 h; (c) knockdown expression of Akt by transfection of siRNA and then treated with Aβ for 24 h; or (d) treatment with Akt activator for 24 h. Thereafter, proteins were extracted from the cell pellets with a radioimmunoprecipitation assay (RIPA) buffer containing protease inhibitors. The concentrations of the samples were determined using the Bicinchoninic acid (BCA) method in which 20 μg of protein samples per lane were loaded in sodium dodecyl sulfate (SDS)-polyacrylamide gels (10%) for electrophoresis and then transferred to the nitrocellulose membranes. The membranes were blocked with 5% Bovine serum albumin (BSA) in Tris-HCl-buffered saline (TBS-T, 0.1% Tween-20) for 2 h at room temperature. Thereafter, the membranes were incubated with primary antibodies at 4°C overnight, before being washed with TBS-T. Subsequently, the membranes were incubated with secondary antibodies [horseradish peroxidase (HRP)-conjugated] antimouse or antirabbit (Cat.sc-516102 and sc-2357, 1:5,000, Santa Cruz Biotechnology, Dallas, TX, USA) at room temperature for 2 h and prepared for band detection using a chemiluminescence detection system (Bio-Rad Laboratories, Hercules, CA, USA). The following primary antibodies were used: rabbit monoclonal anti-SRPK2 (1:3,000, Abcam, Cambridge, United Kingdom, ab192238); rabbit polyclonal Akt antibody (1:3,000, Santa Cruz Biotechnology, Dallas, TX, USA, Cat.sc-81434); mouse monoclonal phosphorylated-Akt antibody (1:3,000, Santa Cruz Biotechnology, Dallas, TX, USA, Cat. sc-377556); rabbit polyclonal phosphorylated-SRPK2 antibody (Thr492, 1:3,000, Thermo Fisher Scientific; Waltham, MA, USA, Cat. PA5-114646); rabbit monoclonal CD16/32 (1:3,000, Abcam, Cambridge, United Kingdom, ab223200); rat monoclonal CD206 (1:3,000, BioLegend, San Diego, California, USA, 141701); rabbit polyclonal apoptotic chromatin condensation inducer 1 (ACIN1) antibody (1:3,000, MyBioSouce, Cat. MBS126801); and mouse monoclonal β-actin (1:3,000, Santa Cruz Biotechnology, Dallas, TX, USA, Cat.sc-47778) antibody.

### Plasmid Construction and Transfection

Human SRPK2 cDNA (Origene, Rockville, Maryland, USA) was sequenced and subcloned into the pUHD10-3 vector (Addgene; Watertown, MA, USA) in accordance with the manufacturer's instructions. The 293T cells were transfected with a lentiviral vector and pUHD10-3-SRPK2-containing plasmid constructs through the use of a lipofectamine 3000 (Thermo Fisher Scientific; Waltham, MA, USA) transfection reagent for 48 h in accordance with the manufacturer's instructions. The screening was performed with tetracycline. The SRPK2 recombinant lentiviral vectors were harvested from the cell supernatant for further experiments. The target cells were incubated with the vectors and subsequently placed in the standard medium for 24 h and then maintained in a medium containing 5 μg/ml tetracycline for 10 days to facilitate clone selection. Western blotting was performed to verify the interference efficiency.

### Small Interfering RNA Transfection

The cells were seeded (1 × 10^5^ cells/well) into 12-well culture plates and transfected with 40 nM of SRPK2 siRNA, Akt siRNA (Santa Cruz Biotechnology, Dallas, TX, USA), or negative siRNA (Santa Cruz Biotechnology) using Lipofectamine 3000 (Invitrogen; Thermo Fisher Scientific) according to the manufacturer's instructions. At 24 h post-transfection, western blotting was performed to assess transfection efficiency, and the cells were used for subsequent experiments. The BV2 cells with SRPK2 knockdown were then stimulated by Aβ or LPS + IFN-γ, while the BV2 cells with Akt knockdown were stimulated by Aβ.

### Immunofluorescence

The BV2 cells were transfected with recombinant lentiviral vectors to increase the expression of SRPK2 or transfected with SRPK2 siRNA to knockdown the expression of SRPK2, while the controls were transfected with empty vectors or negative siRNA. The BV2 cells were then seeded on coverslips. For the detection of ionized calcium-binding adapter molecule 1 (IBA-1) or arginase 1 (Arg-1), the cells were fixed with 4% formaldehyde for 10 min and blocked for 20 min in PBS containing 1% (w/v) BSA-0.1% (v/v) Triton X-100 at room temperature. A primary antibody [rabbit monoclonal anti-IBA-1 (Abcam, Cambridge, United Kingdom, Cat. ab178847) or rabbit monoclonal anti-Arg-1 (cell signaling, Cat.93668)] was diluted to 1:200 and incubated at 4°C overnight. Subsequently, the cells were incubated for 1 h with a 4-μg/ml goat antirabbit immunoglobulin G (IgG) Fluorescein isothiocyanate (FITC) antibody (Abcam, Cambridge, United Kingdom, Cat. ab6717) at room temperature and then mounted in an antifade mounting medium with 4',6-diamidino-2-phenylindole (DAPI). Images were acquired using a fluorescence microscope at X400 magnification (Olympus Corporation, Tokyo, Japan) and processed using ImageJ software (Bethesda, MD, USA) software. In each group, 50 cells at 5 random different areas were measured.

### Statistics

All the results were representative of three independent experiments performed in triplicate. The data were expressed as the mean ± SEM. All the data were subjected to tests for normality. All the data have passed the Kolmogorov–Smirnov (KS) test for normality, *p* > 0.05. The one-way ANOVA followed by Tukey's *post-hoc* test or the Student's *t*-test was used for the statistical analyses in the program of R environment (version 4.0). Differences were considered significant at *p*-values of < 0.05.

## Results

**Figure 1:**
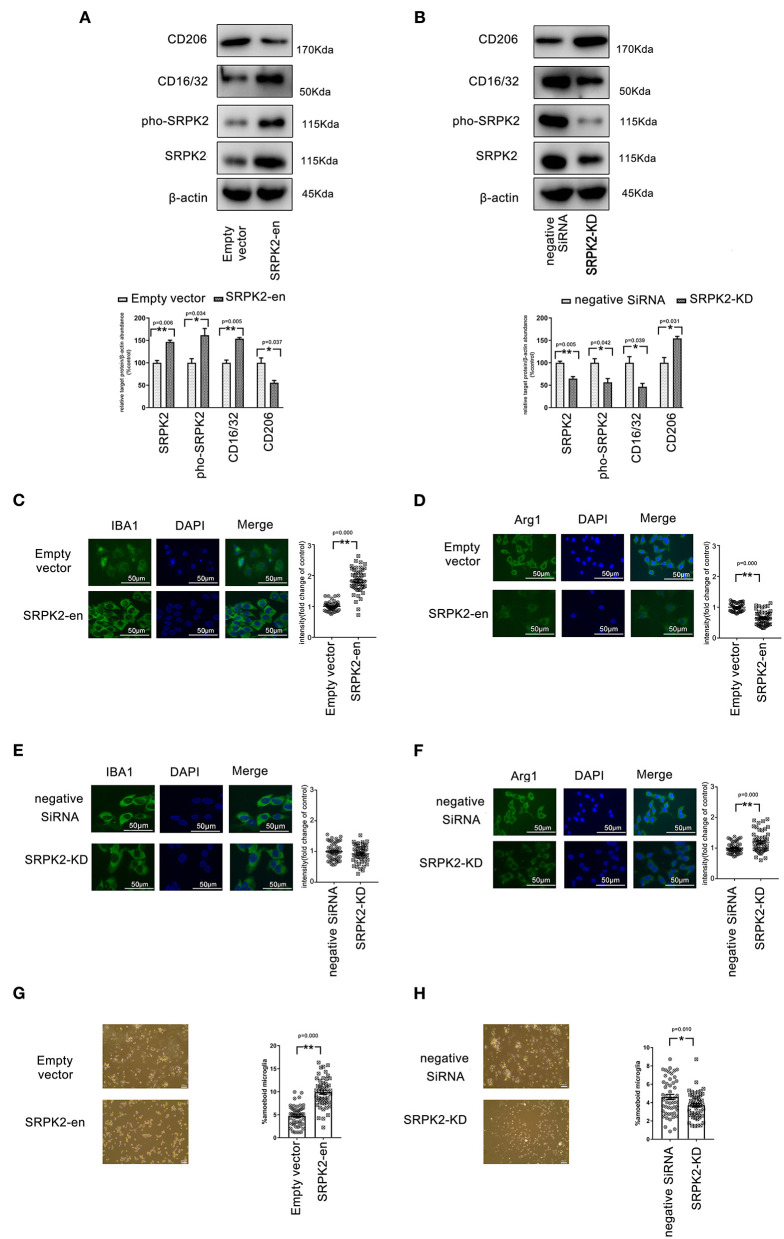
The expression of serine/threonine-protein kinase 2 (SRPK2) contributed to the polarization of the M1 phenotype of the BV2 cells. The transfection of recombinant lentiviral vectors (SRPK2-en) increased the SRPK2 expression and the phosphated SRPK2 level in the BV2 cells compared to the transfection of empty vectors **(A)**, while the knockdown of SRPK2 by the transfection of small interfering RNA (siRNA) (SRPK2-KD) inhibited SRPK2 expression and the phosphated SRPK2 level in the BV2 cells compared to the transfection of negative siRNA **(B)**. Representative images are shown. The data were determined by western blotting and quantitative analysis. The CD206 and CD16/32 protein levels in the BV2 cells were determined by western blot analysis. The expression of SRPK2 by transfection of recombinant lentiviral vectors in the BV2 cells enhanced the expression of CD16/32 and decreased the expression level of CD206 compared to the control cells' transfection of empty vectors **(A,B)**. Conversely, the BV2 cells with SRPK2 knockdown had significantly lower levels of CD16/32 and higher levels of CD206 compared to the control cells transfected with negative siRNA **(A,B)**. The expression levels of ionized calcium-binding adapter molecule-1 (IBA-1) (M1) and arginase-1 (Arg-1) (M2) were measured by immunofluorescence staining **(C–H)**. The BV2 cells were fixed, blocked, and stained with the antibodies of IBA-1 and Arg-1, respectively. The cells were counterstained with DAPI and fluorescence images were acquired under a fluorescence microscope. The quantitative analysis was performed using ImageJ software. The data showed that the BV2 cells with SRPK2 expression had a higher level of expression of IBA-1 **(C)**. However, SRPK2 expression inhibited the expression of Arg-1 in the BV2 cells **(D)**. The BV2 cells with SRPK2 expression knockdown had a higher expression of Arg-1 compared to the cells transfected with negative siRNA **(F)**. **(G,H)** The histogram shows the percentage of ameboid microglia with/without SRPK2 expression. All the results were representative of three independent experiments performed in triplicate. The data were presented as the mean ± SEM of three replicates (*p < 0.05, **p < 0.01 vs. control by the Student's *t*-test).

In previous studies, we demonstrated the increased expression of SRPK2 in the brains of AD mice (Yao et al., 2019). To investigate the effect of SRPK2 in microglial cells, we enhanced the expression of SRPK2 by transfection of recombinant lentiviral vectors or blocking the expression of SRPK2 by siRNA (Figure 1).

### Expression of SRPK2 Contributed to the Polarization of the M1 Phenotype in the BV2 Cells

Activated microglia are often classified into inflammatory (M1) phenotypes, characterized by the production of inflammatory cytokines and expression of IBA-1, while the alternatively activated (M2) phenotypes are characterized by the production of anti-inflammatory cytokine and increased expression of Arg-1.

To investigate the phenotype transformations of microglia, the BV2 cells with/without SRPK2 expression were immune-stained with different biomarkers specific to M1 (CD16/32, IBA-1) or M2 (CD206, Arg-1). As shown in Figure 1, the enhanced expression of SRPK2 increased the expression of CD16/32 and IBA-1, which had lower expression levels of CD206 and Arg-1, in contrast to the cells transfected vectors. However, blocking the expression of SRPK2 by siRNA resulted in decreased CD16/32 and increased CD206 and Arg-1. Thus, the expression of SRPK2 could be related to the activation of microglia with M1 polarization. In addition, morphology analysis showed that enhanced expression of SRPK2 produced a higher percentage of ameboid microglia than that in the controls while blocking the expression of SRPK2 by siRNA resulted in a decreased percentage of ameboid microglia (Figures 1G,H). These data implied greater activation of microglia with SRPK2 expression.

### Expression of SRPK2-Induced Expression of Cytokines in the BV2 Cells

**Figure 2:**
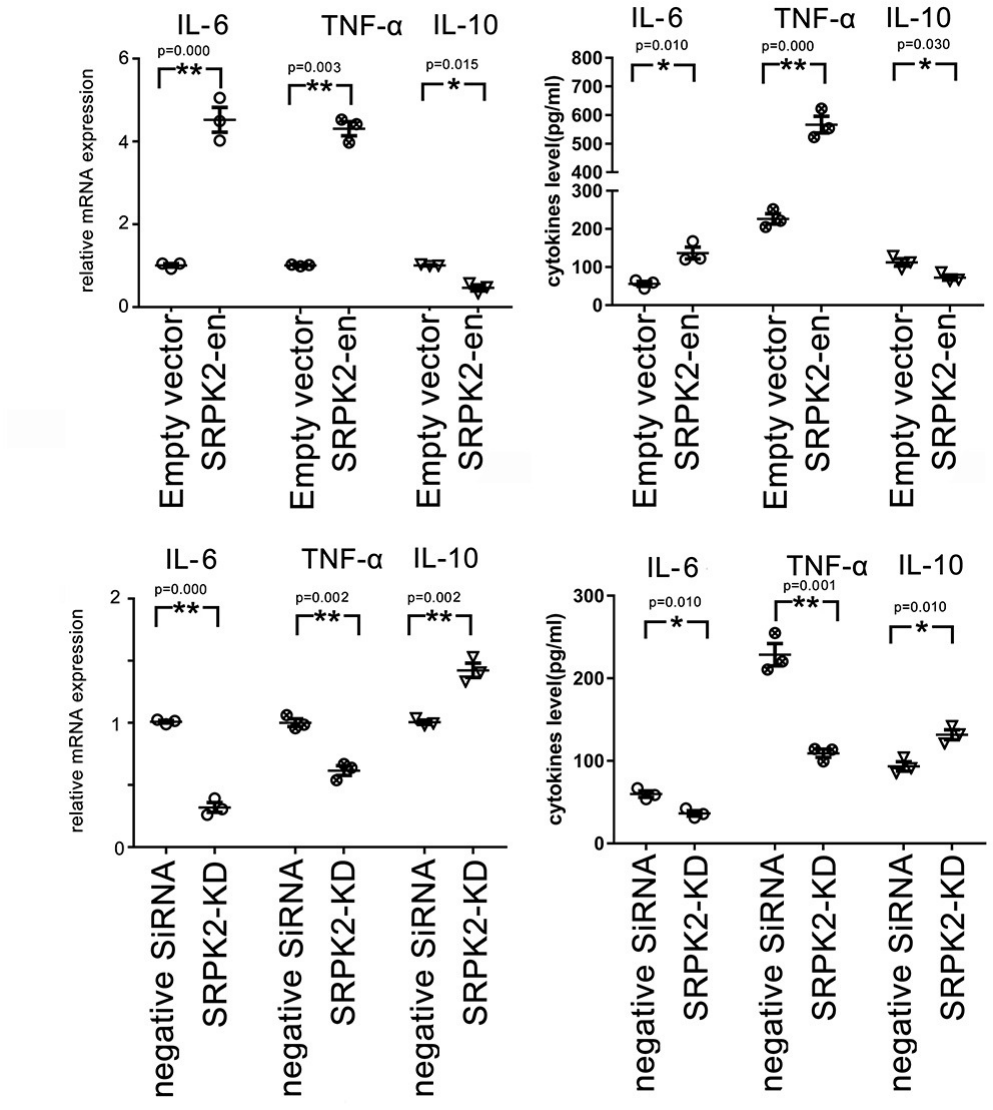
The expression of SRPK2 induces changes in inflammatory cytokines (TNF-α and IL-10) at mRNA and protein levels in the BV2 cells. The expression of SRPK2 was enhanced by the transfection of recombinant lentiviral vectors (SRPK2-en) or knockdown by the transfection of siRNA (SRPK2-KD) in the BV2 cells. The cells and culture medium were collected, respectively. Total RNA was extracted from cells and the mRNA levels of the cytokines of the cells were examined by real-time qPCR. The cytokine levels in the culture medium were examined by ELISA. Our data showed that the cells transfected with SRPK2 recombinant lentiviral vectors had higher expression and secretion levels of IL-6 and TNF-α, but lower levels of IL-10 at mRNA and protein levels compared to the cells transfected with empty vectors. The BV2 cells that were transfected with SRPK2 siRNA had lower expression and secretion levels of IL-6 and TNF-α, but higher levels of IL-10 at mRNA and protein levels compared to the cells that were transfected with negative siRNA. All the results were representative of three independent experiments performed in triplicate. The data were presented as the mean ± SEM of the three replicates (*p < 0.05, **p < 0.01 vs. the control by the Student's *t*-test). SRPK2-en, transfection increased SRPK2 expression; SRPK2-KD, knockdown of SRPK2 by siRNA; TNF-α, tumor necrosis factor-α; IL-10, interleukin-10; IL-6, interleukin-6; mRNA, messenger RNA; qPCR, quantitative PCR.

Besides the expression of CD16/32 or IBA-1, another important feature of the M1 phenotype transformation of microglia is the expression of proinflammatory cytokines. In this study, the BV2 cells received different treatments, after which they were collected, and the messenger RNA (mRNA) levels of cytokines were evaluated by real-time qPCR, while the cytokine levels in the culture medium were evaluated by ELISA. Our data showed that the BV2 cells with SRPK2 expression enhancement had much higher levels of proinflammatory cytokines IL-6 and TNF-α, but lower levels of anti-inflammatory cytokine IL-10, at both the mRNA level and protein level, compared to those in cells without SRPK2 expression enhancement. In contrast, the BV2 cells with SRPK2 expression knockdown had significantly lower levels of proinflammatory cytokines IL-6 and TNF-α, but higher levels of anti-inflammatory cytokine IL-10, at both the mRNA level and protein level, compared to those in cells without SRPK2 expression knockdown (Figure 2).

### Effects of SRPK2 on Cell Viability and Cell Cycle

**Figure 3:**
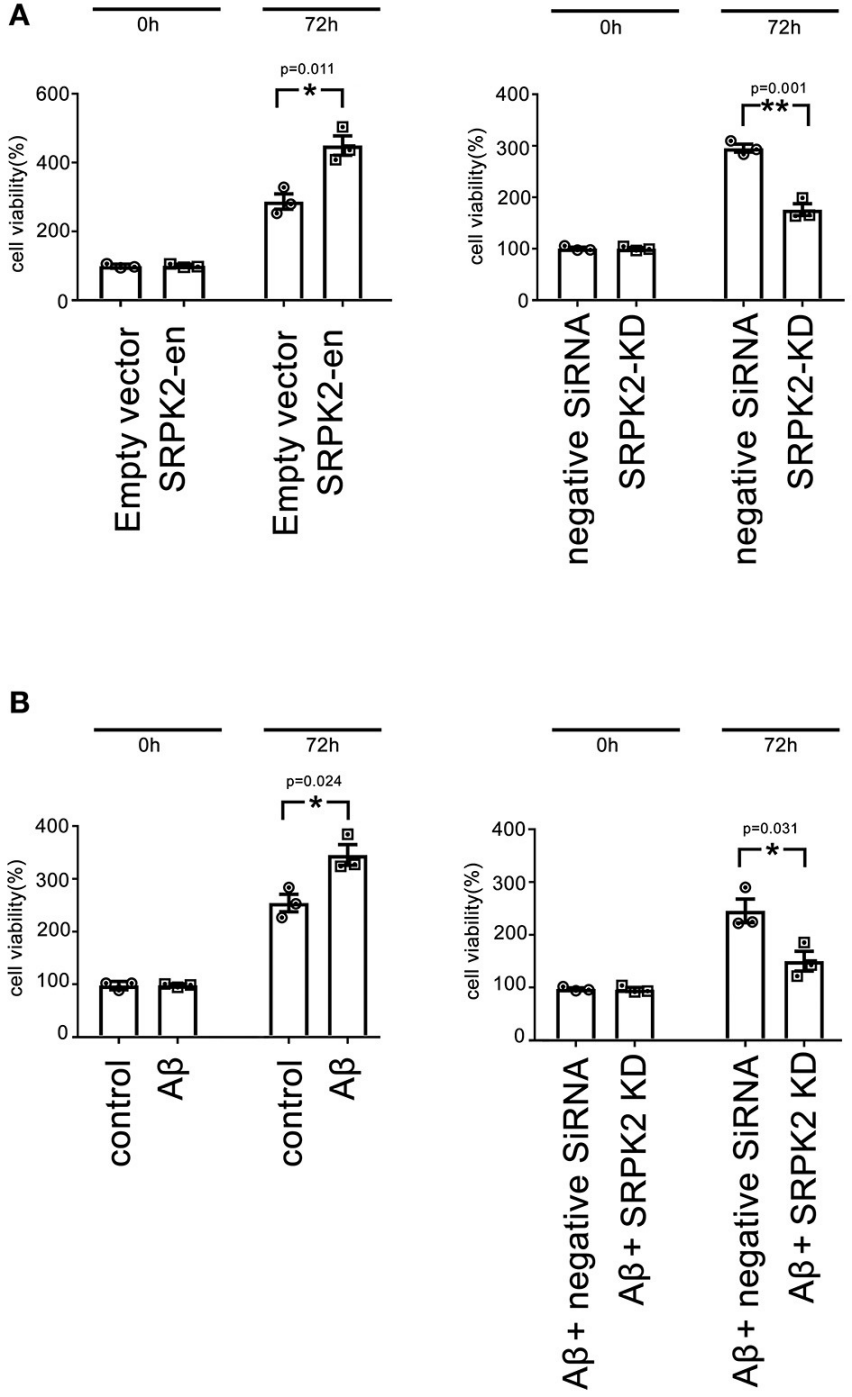
The expression of SRPK2 can promote BV2 cell viability **(A)**. The BV2 cells with different pretreatments (transfection of SRPK2 recombinant lentiviral vectors or knockdown of SRPK2 by transfection of siRNA) were cultured *in vitro* for 72 h and the cell viability was measured by WST-1 assay. The results showed that the cells with the expression of SRPK2 had higher cell viability rates compared to the controls (empty vector transfection), whereas the knockdown SRPK2 inhibited cell viability compared to that in cells transfected with negative siRNA. **(B)** β-amyloid (Aβ) treatment can promote BV2 cell viability, which can be attenuated by the knockdown of SRPK2. The BV2 cells with/without SRPK2 knockdown were treated with Aβ as described in the Methods section for 72 h and the cell viability was measured by WST-1 assay. The results showed that Aβ treatment can promote BV2 cell viability compared to the controls (cells treated with DMSO), whereas the proviability effect of Aβ can be alleviated by knockdown of SRPK2 expression in the BV2 cells. All the results were representative of three independent experiments performed in triplicate. The results were expressed as the mean ± SEM for three replicate determinations (*p < 0.05, **p < 0.01 by the Student's *t*-test). SRPK2-en, transfection increased SRPK2 expression; SRPK2-KD, knockdown of SRPK2 by siRNA; DMSO, dimethyl sulfoxide.

**Figure 4:**
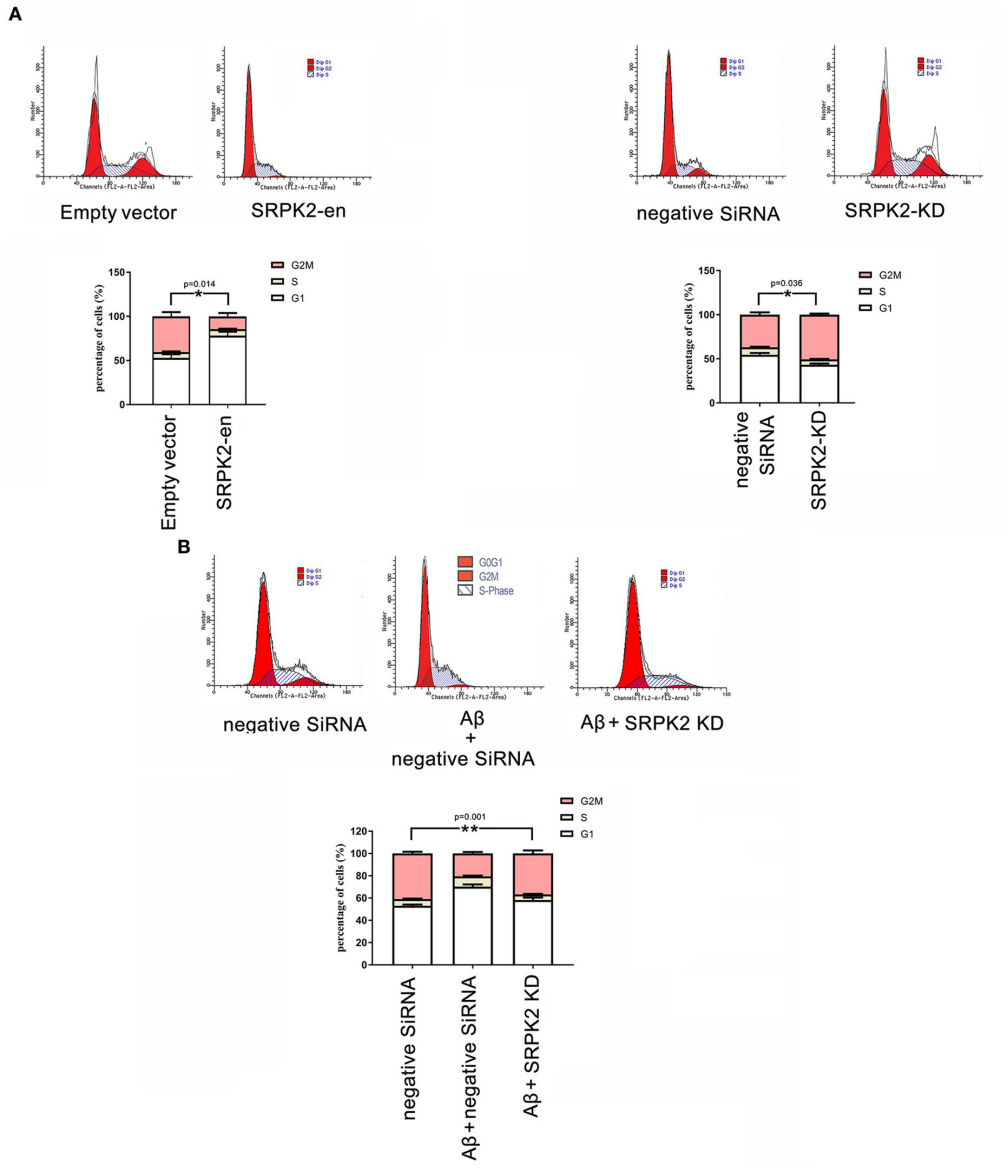
The effect of SRPK2 expression on the BV2 cell cycles. **(A)** The BV2 cells with different treatments (transfection of SRPK2 recombinant lentiviral vectors or knockdown of SRPK2 by transfection of siRNA) were cultured *in vitro* for 72 h. The cells were collected, stained with PI, and analyzed by FACS. The number of cells was indicated as the percentage of different cell cycles. The results showed that the cells with expression of SRPK2 had a higher percentage of G1/S phase compared to the controls (empty vector transfection), whereas the knockdown SRPK2 had a lower percentage of G1/S phase compared to cells transfected with negative siRNA. **(B)** Aβ treatment increased the percentage of the G1/S phase of the BV2 cells, which can be attenuated by the knockdown of SRPK2. The BV2 cells with SRPK2 knockdown were treated with Aβ as described in the Methods section for 72 h and the cell cycles were measured and analyzed by FACS. All the results were representative of three independent experiments performed in triplicate. The results were expressed as the mean ± SEM for three replicate determinations (*p < 0.05, **p < 0.01 by the Student's *t*-test or one-way ANOVA). SRPK2-en, transfection increased SRPK2 expression; SRPK2-KD, knockdown of SRPK2 by siRNA.

The BV2 cells received different treatments including the transfection of recombinant lentiviral vectors to enhance SRPK2 expression or inhibition of the expression of SRPK2 by transfection of siRNA. Thereafter, the cells were cultured *in vitro* for 72 h. The results showed that expression enhancement of SRPK2 clearly increased cell viability, while the cells with SRPK2 knockdown had lower cell viability (Figure 3A). Also, the percentage of the G1/S phase increased in the cells with SRPK2 expression enhancement, while the knockdown of SRPK2 decreased the percentage of the G1/S phase of the BV2 cells (Figure 4A).

### β-Amyloid Promoted the Viability of Microglia Through SRPK2 Expression

**Figure 5:**
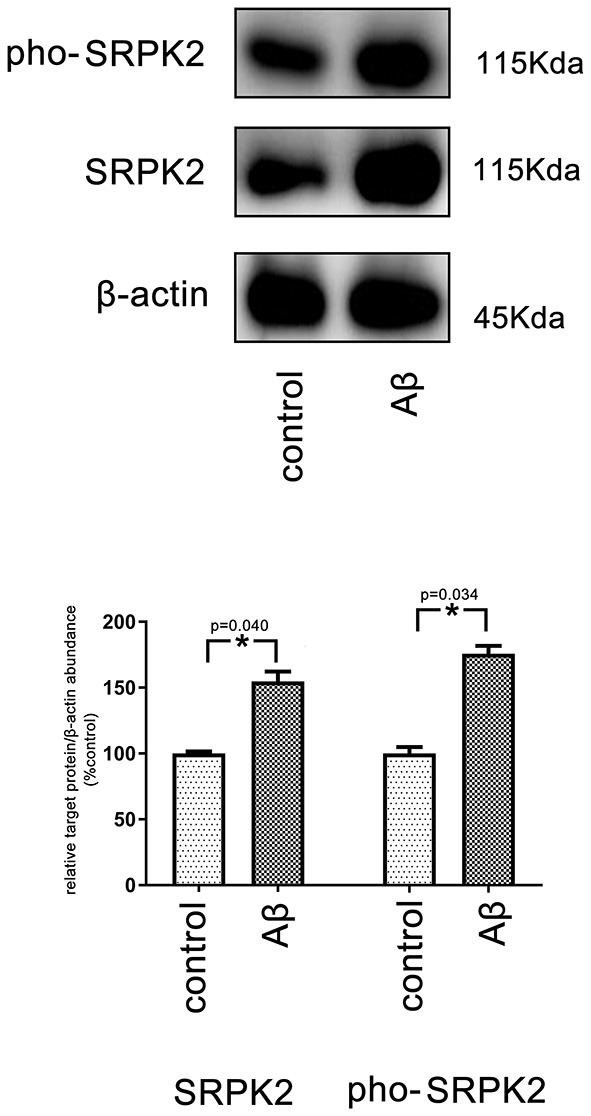
The expression level of SRPK2 in the BV2 cells after treatment with Aβ. The BV2 cells were treated with Aβ for 24 h, while the cells treated with DMSO were considered as controls. The cells were collected and the expression of SRPK2 and phosphated SRPK2 levels were examined by the western plot. The results showed that the expression of both the SRPK2 level and the phosphated SRPK2 level was significantly increased compared to the controls. All the experiments were performed independently in triplicate. The data were presented as the mean ± SEM. *p < 0.05, by the Student's *t*-test. pho-SRPK2, phosphated SRPK2.

It is known that treatment with Aβ can activate microglia. In this study, Aβ treatment promoted BV2 cell viability with a high G1/S phase percentage. However, the proviability effect of Aβ can be attenuated by blocking SRPK2 expression (Figures 3B, 4B). In addition, we used Aβ to treat the BV2 cells *in vitro*. The data showed that treatment with Aβ for 24 h increased the expression of SRPK2 in the BV2 cells (Figure 5). Therefore, we speculated that Aβ might regulate the activity of microglia cells through the expression of SRPK2.

### Serine/Threonine-Protein Kinase 2 Deficiency Attenuated the Toxic Effect of Microglia on Cortical Neurons

**Figure 6:**
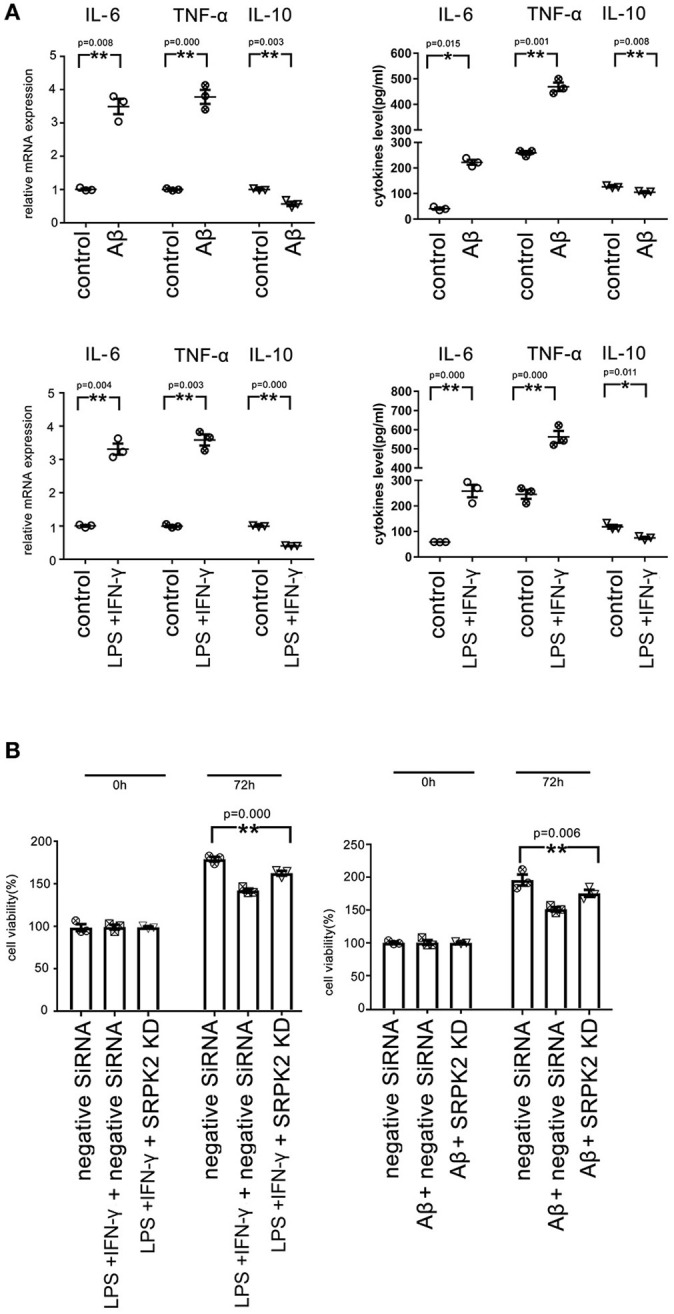
Both the Aβ and LPS + IFN-γ can promote the cytotoxicity of the BV2 cells *via* the expression of SRPK2. The BV2 cells with/without SRPK2 expression (by transfection of SRPK2 recombinant lentiviral vectors or knockdown of SRPK2 by transfection of siRNA) were stimulated by Aβ or LPS + IFN-γ for 24 h, while the control cells were treated with DMSO (control for Aβ) or PBS (control for or LPS + IFN-γ). The cells and culture medium for each were collected. **(A)** The total RNA was extracted from the cells and the mRNA levels of the cytokines of the cells were examined by real-time qPCR. The cytokine levels in the culture medium were examined by ELISA. The results showed that treatment with LPS + IFN-γ or Aβ can increase the levels of IL-6 and TNF-α, but decrease the level of IL-10 at mRNA and protein levels compared to the controls, respectively. **(B)** The culture medium was collected and used to treat HT-22 cells for 48 h. Cell viability of HT-22 cells was evaluated by the WST-1 assay. Our data showed that the BV2 cells stimulated by Aβ or LPS + IFN-γ exerted significant cytotoxicity on HT-22 cells by inhibiting cell viability compared to the controls, respectively. However, knockdown of the expression of SRPK2 in the BV2 cells could alleviate the cytotoxicity of the BV2 cells induced by Aβ or LPS + IFN-γ, in which the HT-22 cells exhibited higher cell viability when treated with culture medium derived from the SRPK2-knockdown BV2 cells compared to that from the BV2 cells transfected with negative siRNA. All the results represented three independent experiments performed in triplicate. The results were expressed as the mean ± SEM for three replicate determinations (*p < 0.05, **p < 0.01 by the Student's *t*-test or one-way ANOVA). SRPK2-KD, knockdown of SRPK2 by siRNA; LPS, lipopolysaccharide; IFN-γ, interferon-γ; PBS, phosphate-buffered saline.

We found that both the LPS + IFN-γ and Aβ treatment led to increased levels of IL-6 and TNF-α, but decreased levels of IL-10 in microglia, compared to the cells of the control group (Figure 6A).

It has been demonstrated that activated microglia can mediate injury of neuronal cells (Zhao et al., 2004; Culbert et al., 2006). To investigate the role of SRPK2 in the neuronal cell injury mediated by microglia, we stimulated microglial cells by treatment of LPS + IFN-γ or Aβ, and then the neuronal cells were cultured with a medium derived from stimulated microglial cells. The results showed that the microglial cells stimulated by LPS + IFN-γ or Aβ can inhibit the cell viability of neuronal cells. Furthermore, neuronal cell viability was clearly higher when cultured with a medium derived from stimulated microglia with SRPK2 knockdown compared to a conditioned medium derived from microglial cells without SRPK2 knockdown (Figure 6B).

### Serine/Threonine-Protein Kinase 2 Was Activated by the Akt Pathway in Microglial Cells

**Figure 7:**
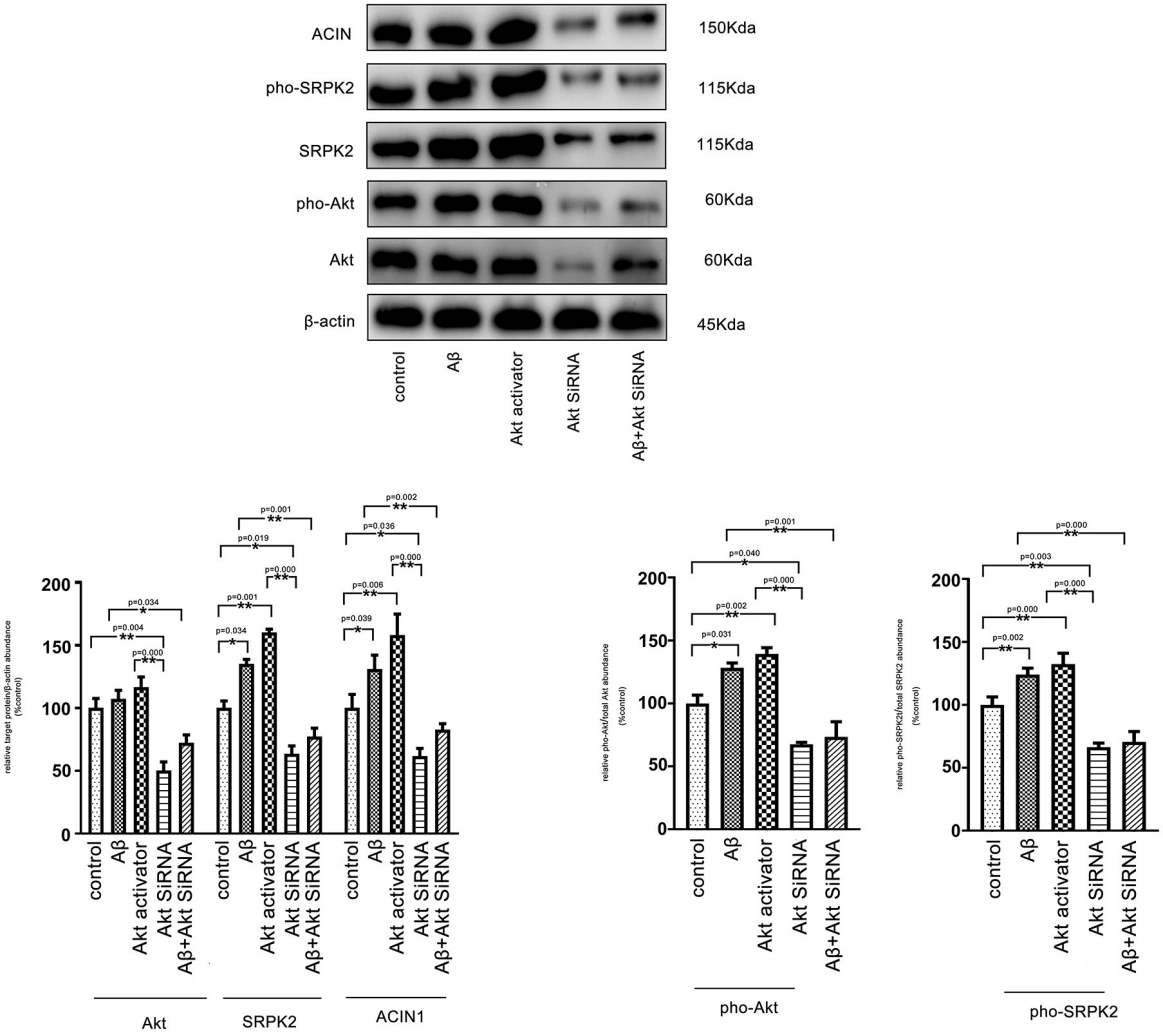
Aβ regulates SRPK2 expression through the Akt pathway. The BV2 cells were treated with Akt activator or Aβ for 24 h *in vitro* and SRPK2 expression was measured by western blot. The results showed that both the Aβ and Akt activator can increase the expression of SRPK2 level and phosphated SRPK2 level and ACIN1 level. However, when the expression of Akt was knockdown by siRNA in the BV2 cells, the regulatory effect of Aβ on the expression of SRPK2 and its related pathway was abolished including the expression of SRPK2 level and phosphated SRPK2 level and ACIN1 level. All the results were representative of three independent experiments performed in triplicate. The data were shown as the mean ± SEM (*p < 0.05; **p < 0.01 by the one-way ANOVA). Akt siRNA, knockdown of Akt by siRNA; pho-Akt, phosphated Akt; pho-SRPK2, phosphated SRPK2.

It is known that Aβ-mediated changes can result in phosphorylation of Akt and contribute to AD pathogenic events in microglia (Zhou et al., 2008). ACIN1 is a protein downstream of the SRPK2 pathway; SRPK2 can phosphorylate ACIN1 and redistribute nucleoplasm, resulting in cyclin A1 upregulation (Jang et al., 2008). We investigated the potential interaction between the Akt pathway and SRPK2. Our results indicated that both the Aβ and Akt activator significantly increased the expression of SRPK2 levels and phosphorylated SRPK2 and ACIN1 levels in the BV2 cells. Blocking of Akt by siRNA decreased the expression of SRPK2 level and phosphorylated SRPK2 and ACIN1 levels. However, in the BV2 cells with Akt deficiency, the promoting effect of Aβ on SRPK2 was clearly blocked (Figure 7).

## Discussion

This study shows that SRPK2 is involved in microglia-mediated neurotoxicity induced by Aβ. Aβ can promote the expression of SRPK2, which can contribute to the activation of the microglial cell line, BV2. The expression of SRPK2 is related to the production of proinflammatory cytokines, viability, and M1 polarization of microglial cells. Moreover, the expression of SRPK2 could be attributed to the activated pathway of Akt.

Serine/threonine-protein kinase 2 belongs to the Serine/Arginine-Rich Protein-Specific Kinase family (SRKF) family, including multiple SRPKs1–3, some of which are expressed in immune cells and regulate the inflammatory response. The expression of SRPK2 is found in the central nervous system. Sebastian Monasor et al. (2020) identified the expression of SRPK2 in microglia in AD mice and we found that the increased expression of SRPK2 is related to the decline of cognitive ability of the AD mice model. Hong et al. (2012) have revealed that SRPK2 contributes to AD pathology through the regulation of phosphorylation of tau protein. In this study, we found that SRPK2 could be involved in the activation of microglia cells, contributing to AD pathology.

It has been known for many years that Aβ plays a key role in AD pathology in multiple ways including inducing an inflammatory response in the brain through interaction with microglial cells. In recent years, the effect of microglia in AD pathology has drawn the attention of researchers. Aβ accumulation in parenchyma and blood vessels causes microglial migration, activation, and polarization, as acute and chronic inflammatory responses against the aggregates, thus inducing the production of nitric oxide (NO), reactive oxygen species, proinflammatory cytokines (TNF-α, IL-1β, and IL-6), chemoattractant protein-1, cyclooxygenase-2 (COX-2), complement component 1q, etc. (Tang and Le, 2016; Tiwari et al., 2019; Sebastian Monasor et al., 2020). Although studies on the effect of inflammatory cytokines on AD pathology are controversial, more and more studies have provided evidence that microglial-derived cytokines can provoke neuronal death. Both IL-1 and IL-6 can promote Amyloid-beta precursor protein (APP) protein expression (Tang and Le, 2016). Moreover, IL-6 can contribute to neurofibrillary tangles formation by inducing tau phosphorylation through CDK5/p35 pathway deregulation (Quintanilla et al., 2004). In addition, microglial-derived TNF-α can induce NO production in neuronal cells, which triggers NO-mediated neuronal apoptosis (Guadagno et al., 2013). However, the mechanism of the M1 phenotype switch of microglial in AD pathology remains unknown. This study reveals that Aβ induced the expression of SRPK2 in microglia, which is involved in the activation of M1 polarization of microglia, with increased production of proinflammatory cytokines. Therefore, we suggested that the regulatory effect of SRPK2 in microglia may be another pathway in AD pathology.

Furthermore, this study observed that Aβ can promote SRPK2 expression via the Akt pathway in microglia. In AD pathology, multiple studies have demonstrated that the activated Akt pathway contributes to the M1 phenotype transformation of microglia induced by Aβ, which causes inflammation and cytotoxicity. A previous study has demonstrated that Akt phosphorylated SRPK2 on Thr-492, in turn, the phosphorylated SRPK2 translocated into nuclear to regulate the cell cycle, DNA synthesis, and apoptosis (Jang et al., 2009). From the foregoing, we believe that SRPK2 could be another downstream node of the Akt pathway in microglia. Therefore, we suggest that Aβ activates the Akt pathway, which may trigger the expression of SRPK2 and the eventual activation of M1 polarization of microglia. In contrast, Aβ failed to regulate the expression of SRPK2 when Akt was knocked down in microglia. To the best of our knowledge, this is the first study that explores the effect of SRPK2 and the potential proinflammatory mechanism of its contribution to AD pathology. Hence, further study is needed to determine the role of SRPK2 in microglia in relation to AD pathology.

In addition, this study suffers from some limitations. We did not involve the animal model or investigate the effect of SRPK2 in the primary microglial cells in this study, which could provide more evidence to verify the effect of SRPK2 in the activation of microglial cells; thus, further investigations are required to understand the role of SRPK2 in activation of microglial cells.

In summary, this study has demonstrated that SRPK2 could be involved in microglia-mediated neurotoxicity, potentiating proinflammatory effects in Aβ-stimulated microglial cells. In addition, we have determined that the SRPK2-related pathway could be associated with the activation of microglial cells in AD pathology as well as the neuroprotective effects of SRPK2 inhibition in the Aβ-activated BV2 cells. Therefore, SRPK2 may be an important modulating pathway for inflammatory mediators in AD pathology. In recent years, several studies have demonstrated that inflammation is another central mechanism in the pathology of AD (Akiyama et al., 2000; Holmes, 2013; Ozben and Ozben, 2019), in addition to Aβ and neurofibrillary tangles, convincing researchers to propose anti-inflammation as a new therapeutic approach for AD treatment. Thus, our findings provide new insights into the AD therapeutic potential of modulating the microglial functional state by targeting SRPK2.

## Data Availability Statement

The original contributions presented in the study are included in the article/Supplementary Material, further inquiries can be directed to the corresponding authors.

## Author Contributions

ZT and WZ performed the cell experiments. CY, QL, and NL participated in performing the animal experiments. LR and JL analyzed the data. XY and SL drafted the manuscript. XY and SL conceived, designed, and coordinated the study. All authors read and approved the final manuscript.

## Funding

This study was supported by the Natural Science Foundation of Hebei Province, China (grant no. H2020423198).

## Conflict of Interest

The authors declare that the research was conducted in the absence of any commercial or financial relationships that could be construed as a potential conflict of interest.

## Publisher's Note

All claims expressed in this article are solely those of the authors and do not necessarily represent those of their affiliated organizations, or those of the publisher, the editors and the reviewers. Any product that may be evaluated in this article, or claim that may be made by its manufacturer, is not guaranteed or endorsed by the publisher.
