# Supplementary material for: SRPK2 Expression and Beta-Amyloid Accumulation Are Associated With BV2 Microglia Activation
Source: Front Integr Neurosci. 2022 Jan 28;15:742377. doi: 10.3389/fnint.2021.742377 (PMC8831369; doi:10.3389/fnint.2021.742377)

Figure 1

First time

β-actin

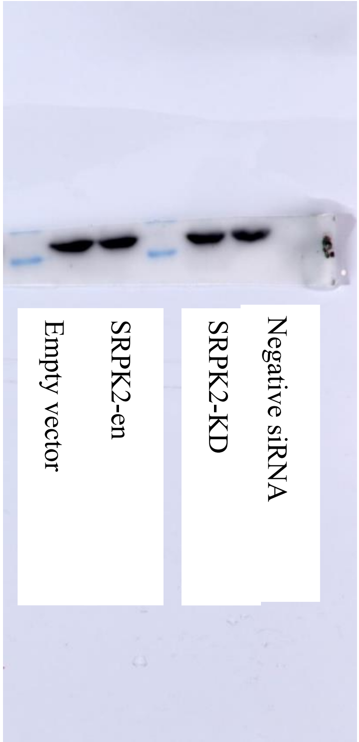

SRPK2

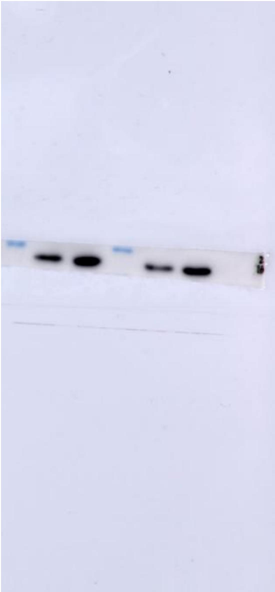

Pho-SRPK2

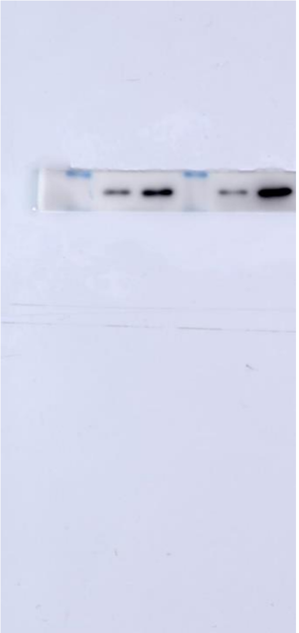

CD16/32

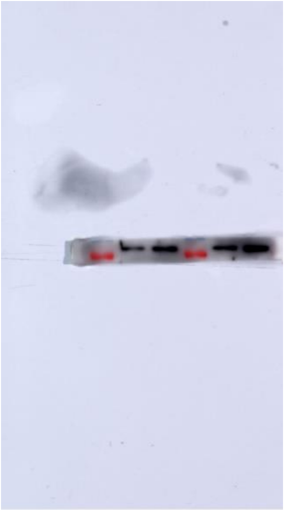

CD206

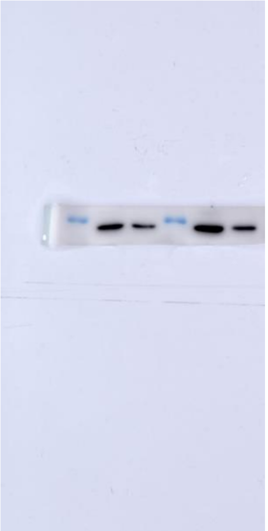

Second time

β-actin

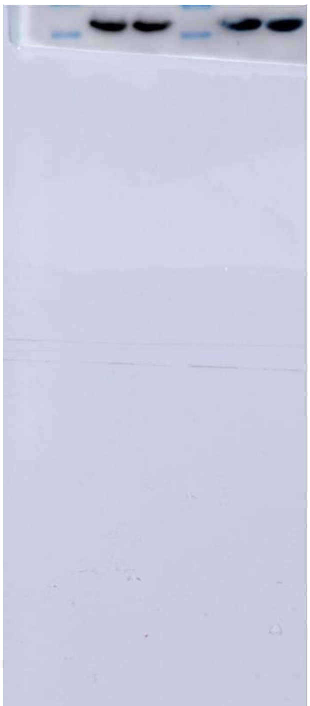

SRPK2

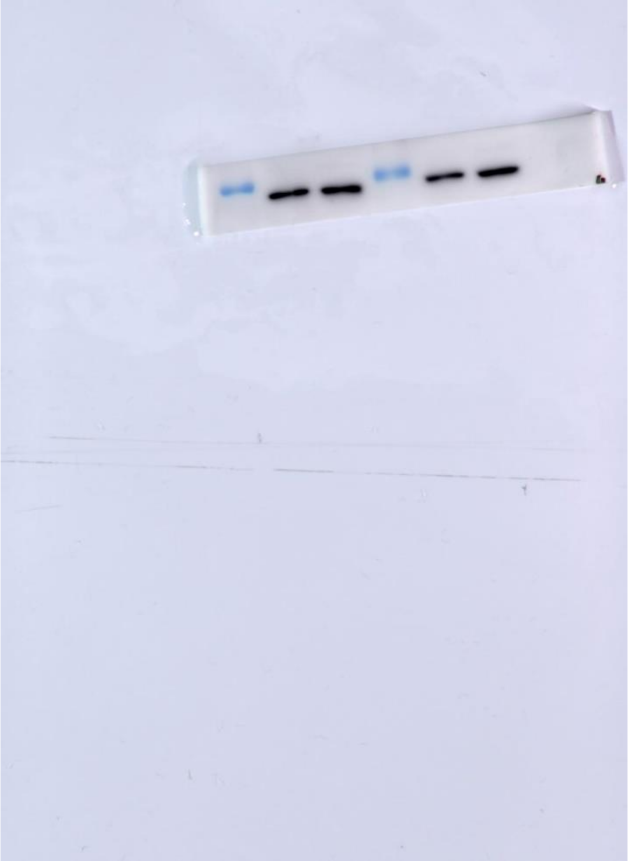

Pho-SRPK2

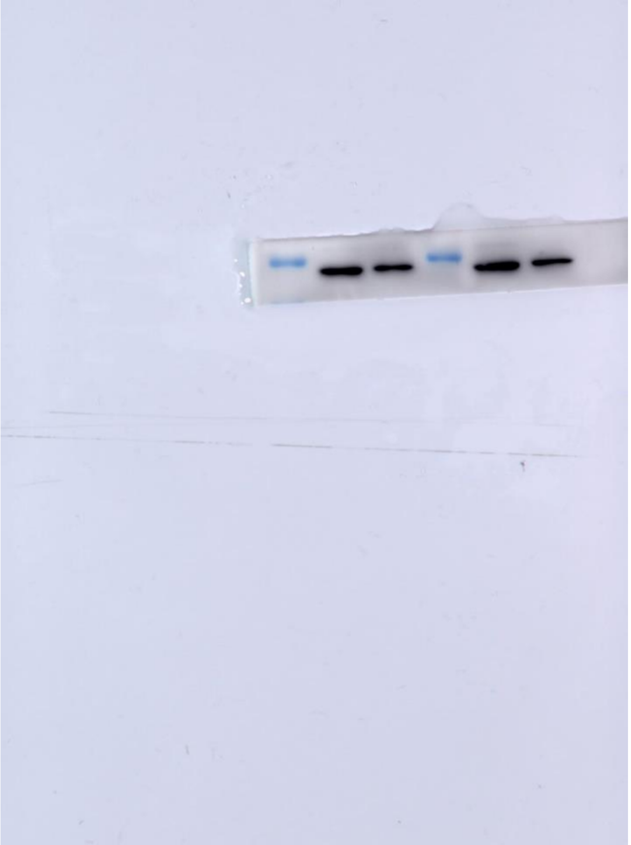

CD16/32

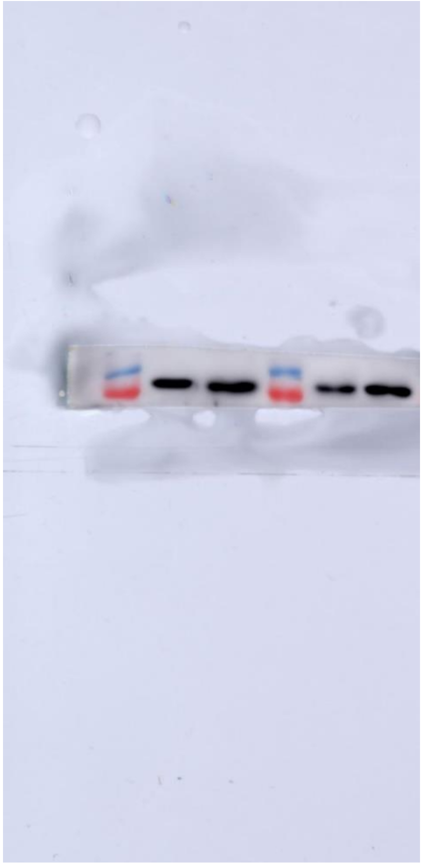

CD206

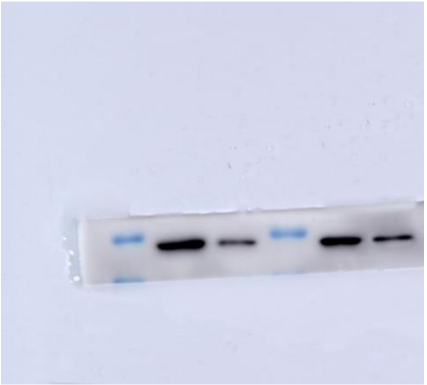

Third time

SRPK2

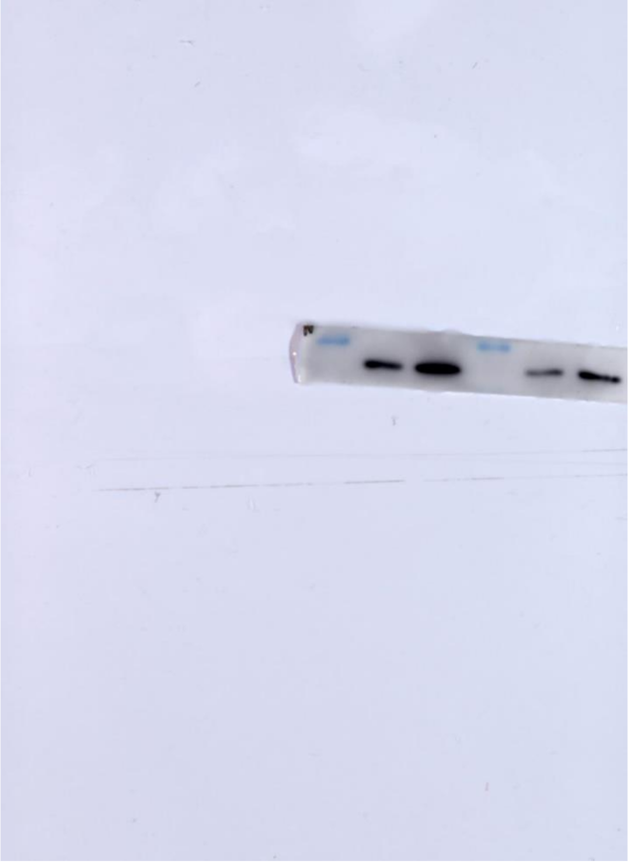

Pho-SRPK2

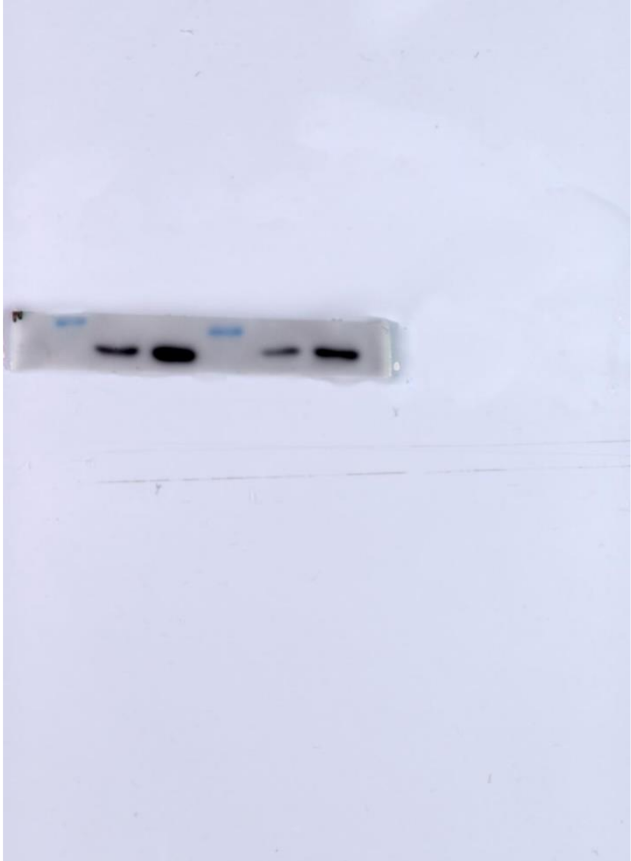

CD16/32

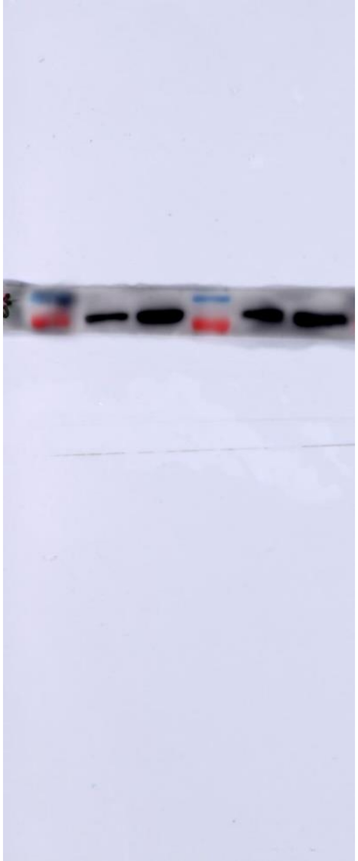

CD206

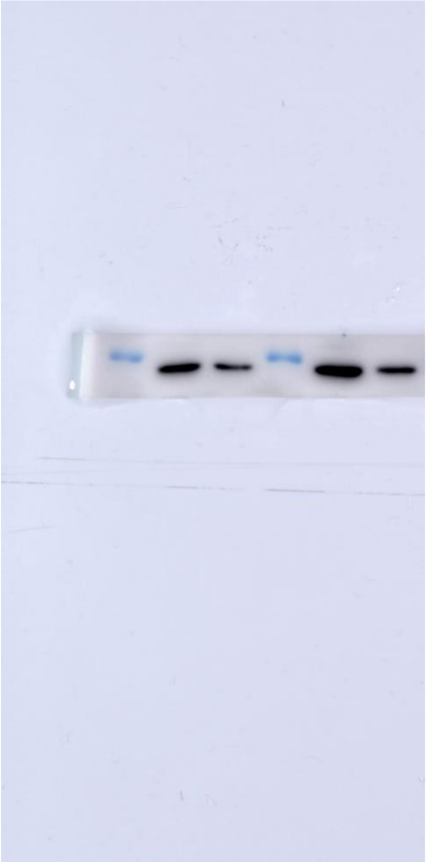

Figure 5

First time

β-ACTIN

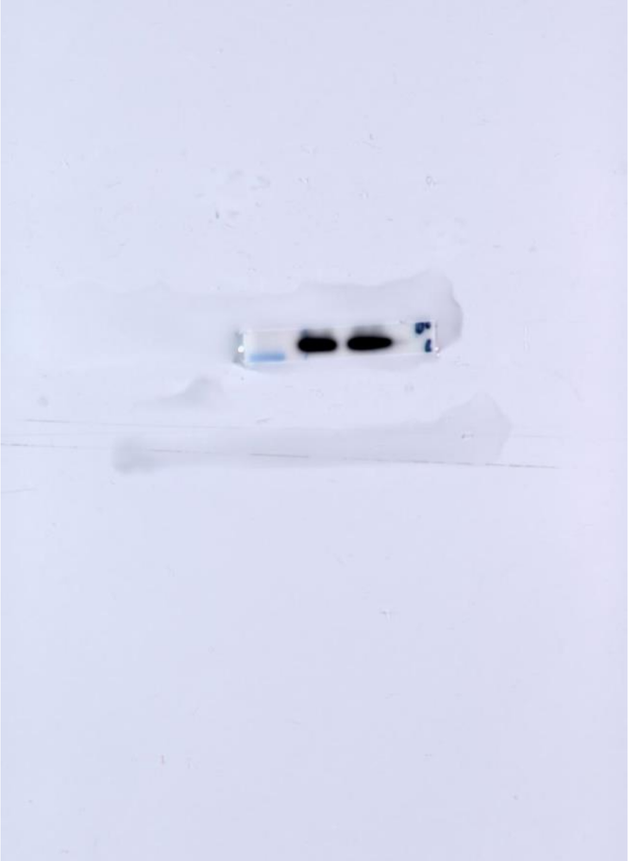

SRPK2

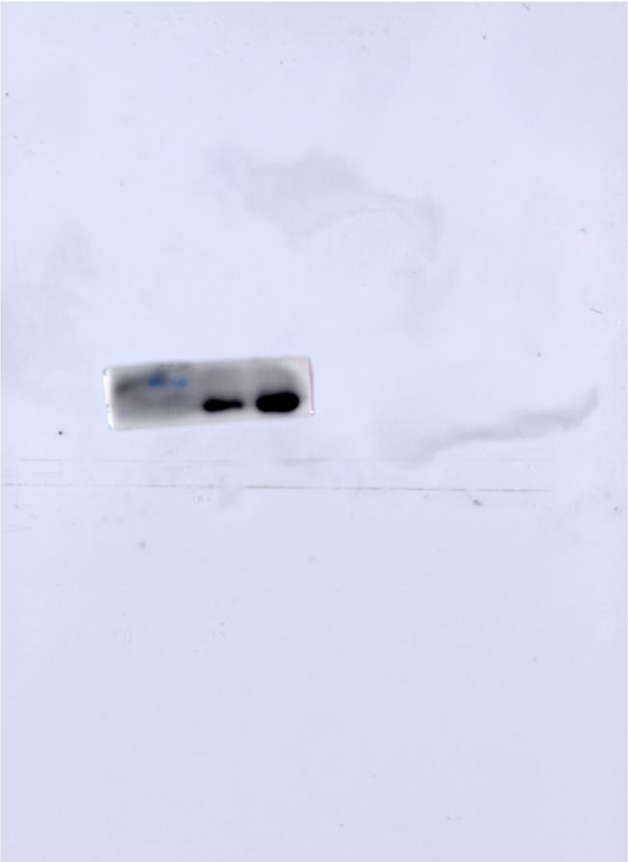

Pho-SRPK2

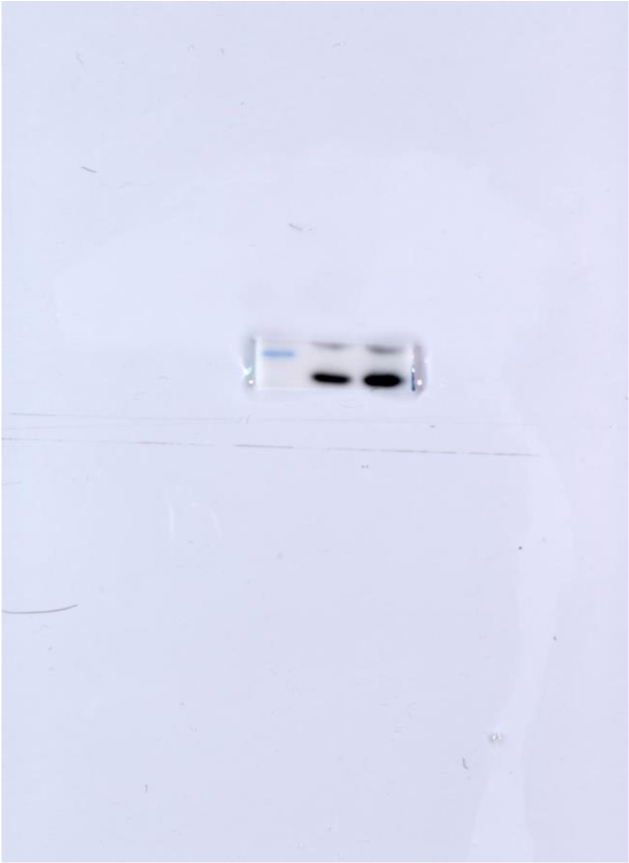

Second time

β-ACTIN

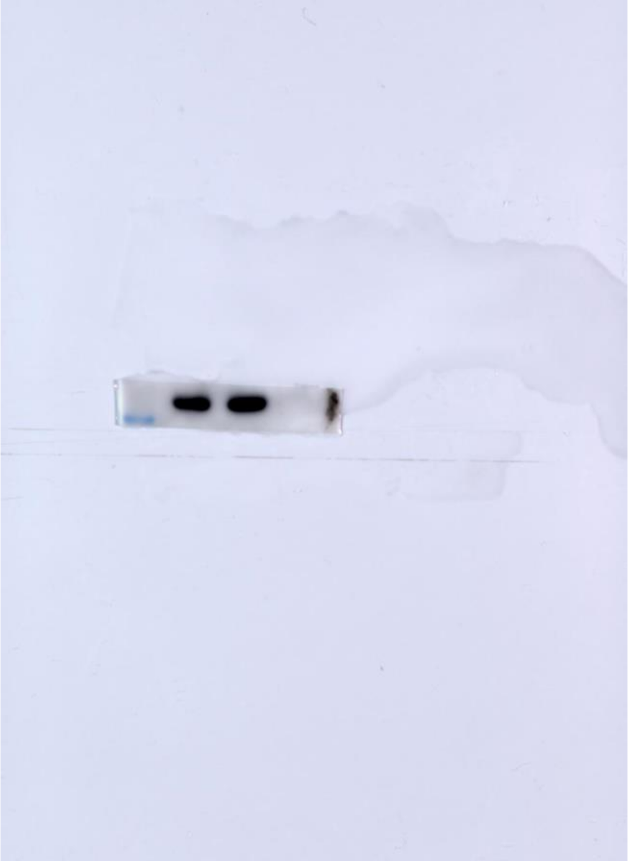

SRPK2

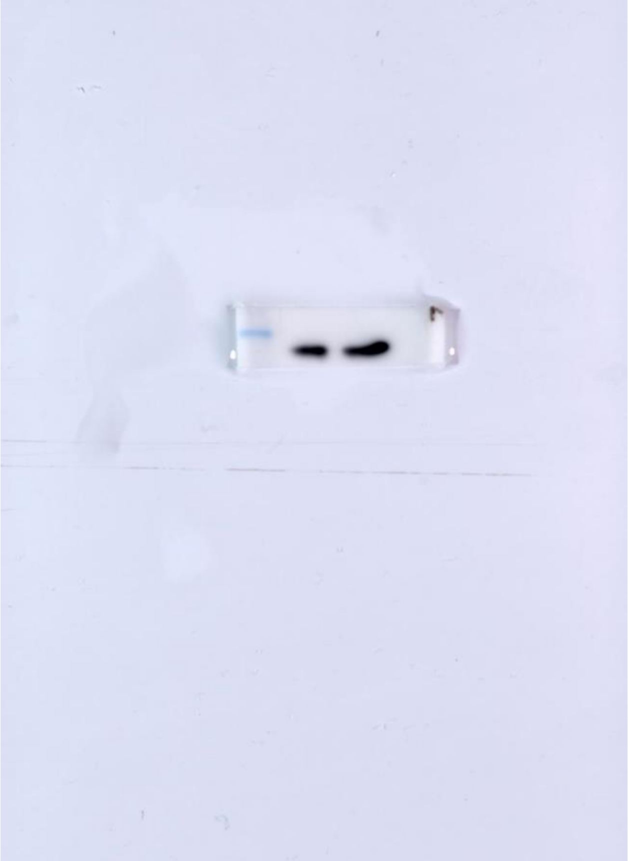

Pho-SRPK2

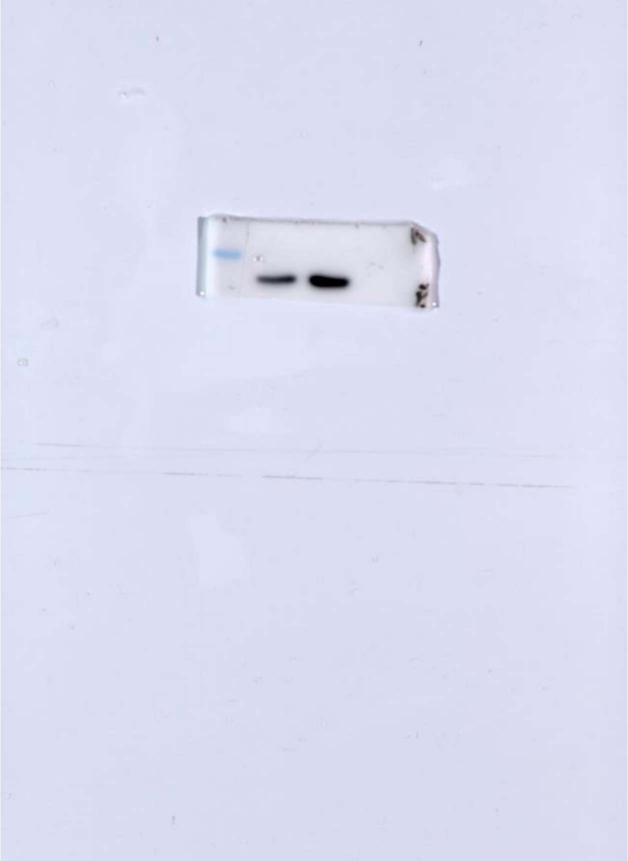

Third time

β-ACTIN

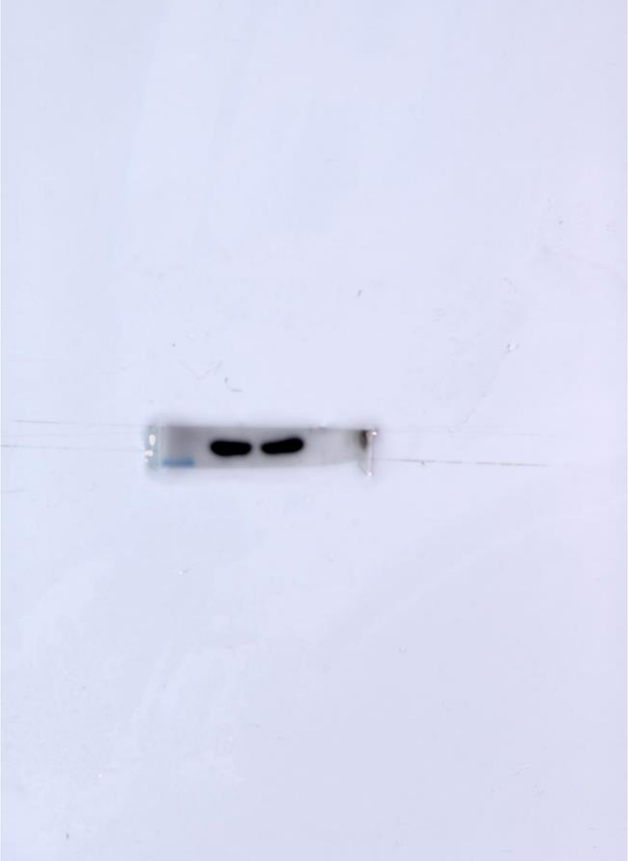

SRPK2

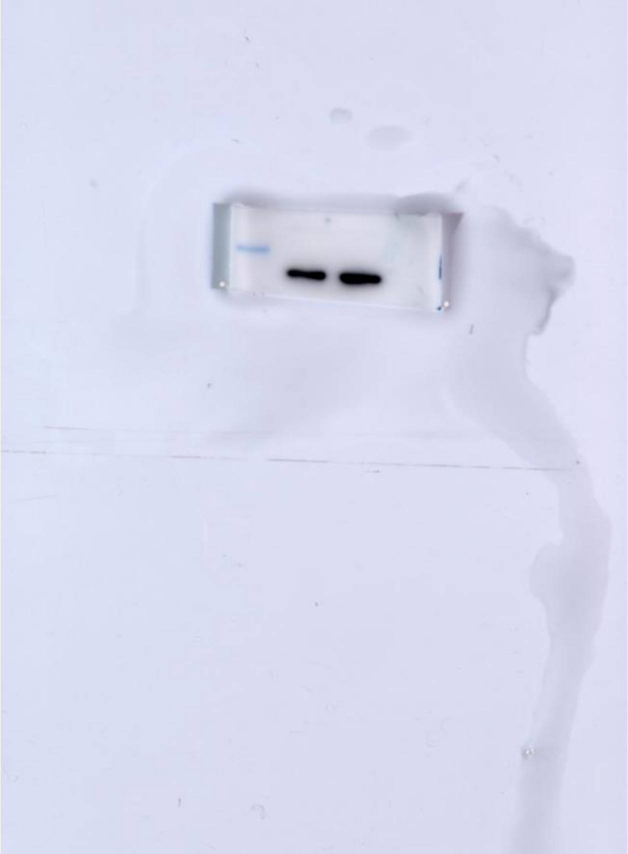

Pho-SRPK2

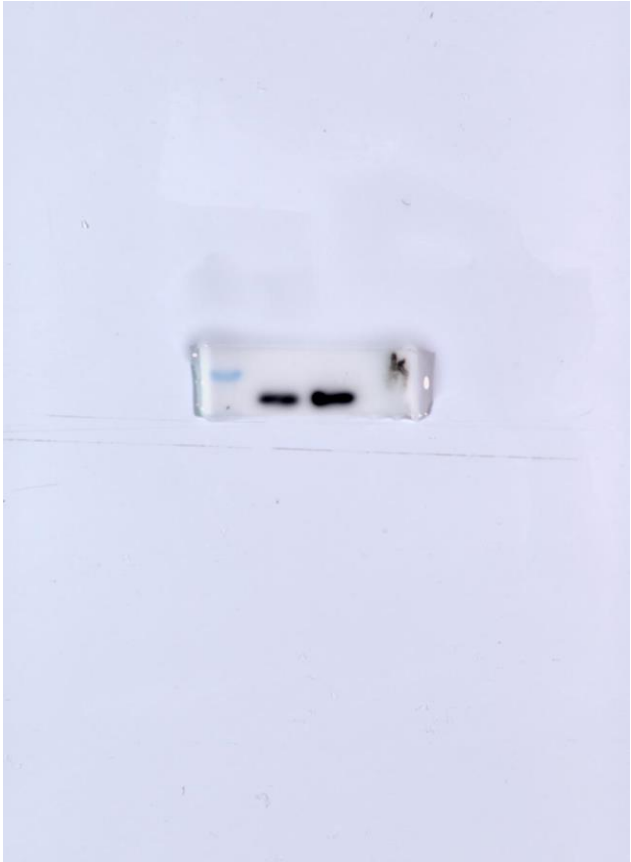

Figure 7

First time

β-ACTIN

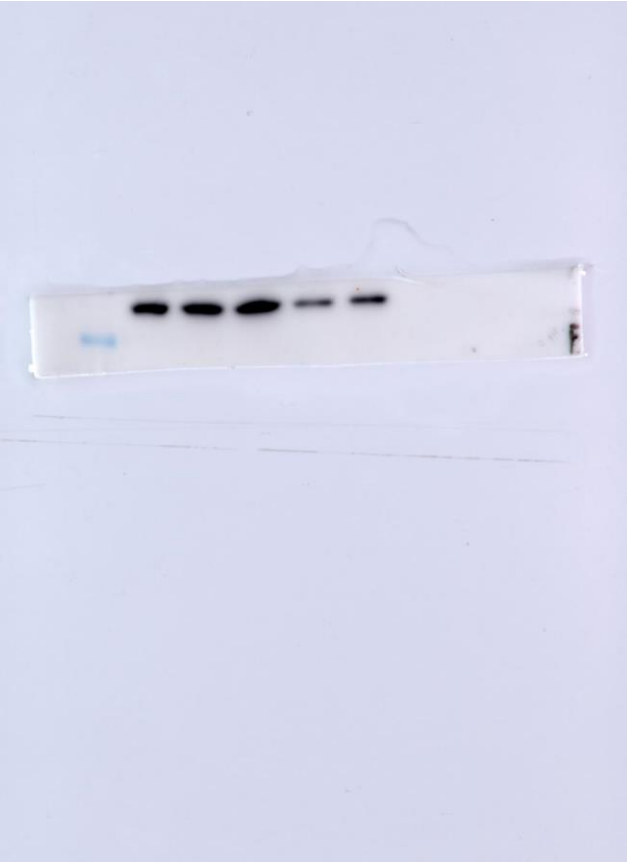

Akt

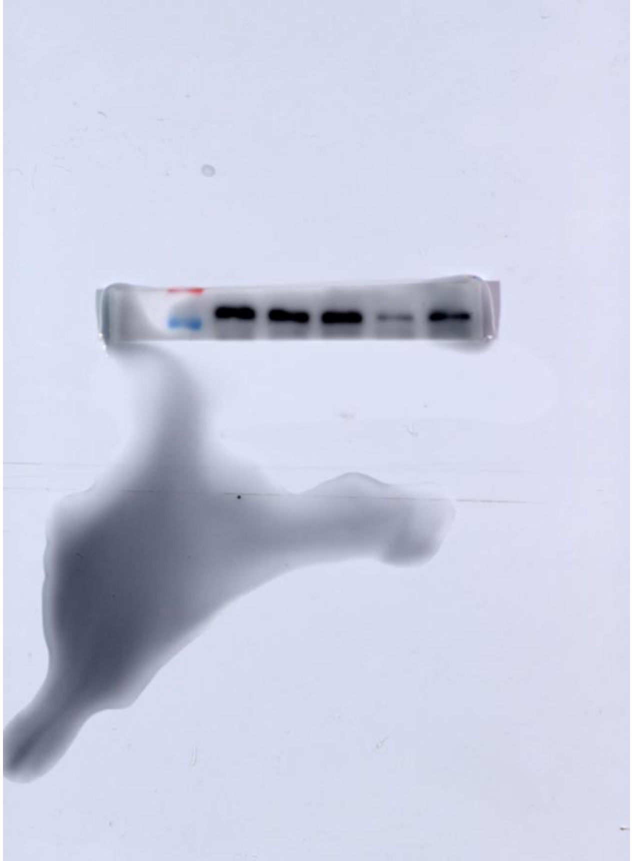

Pho-Akt

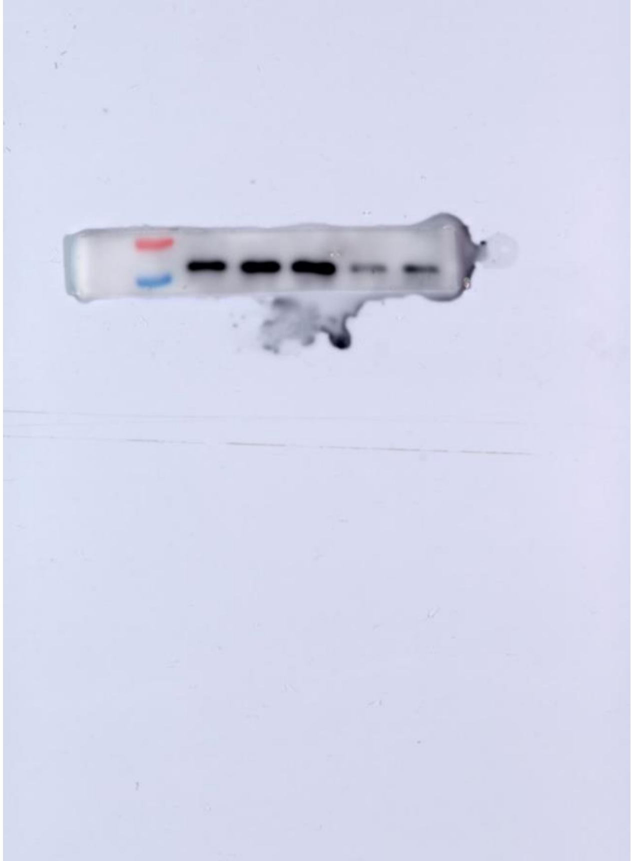

SRPK2

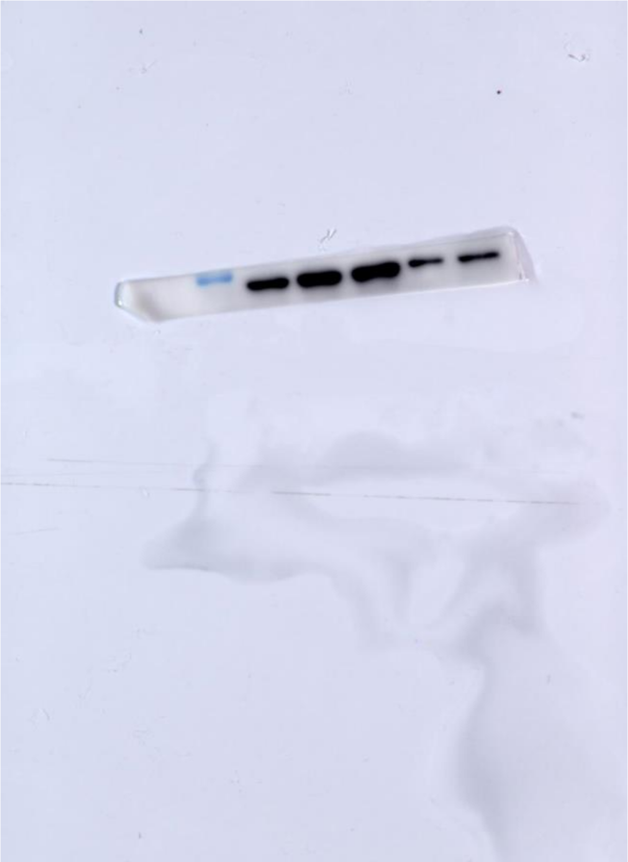

Pho-SRPK2

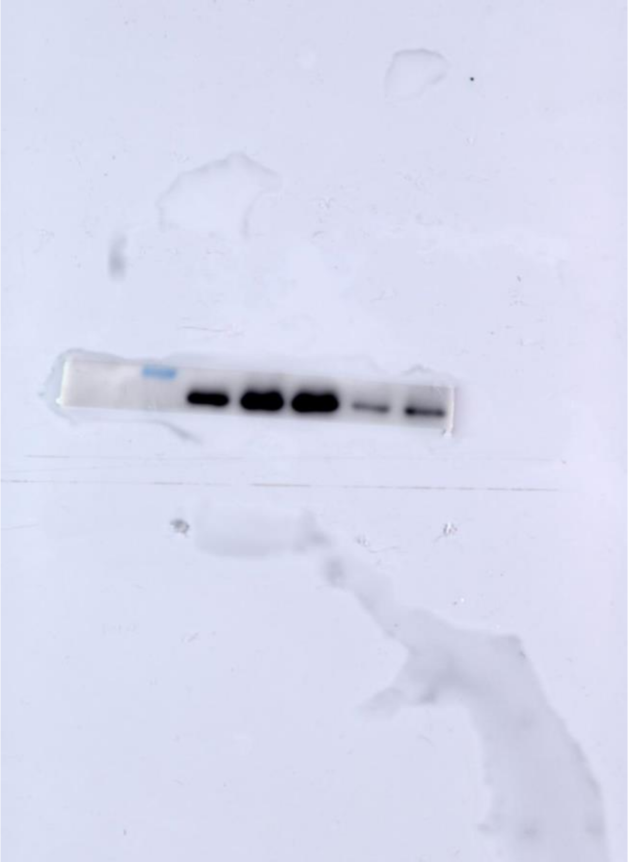

ACIN

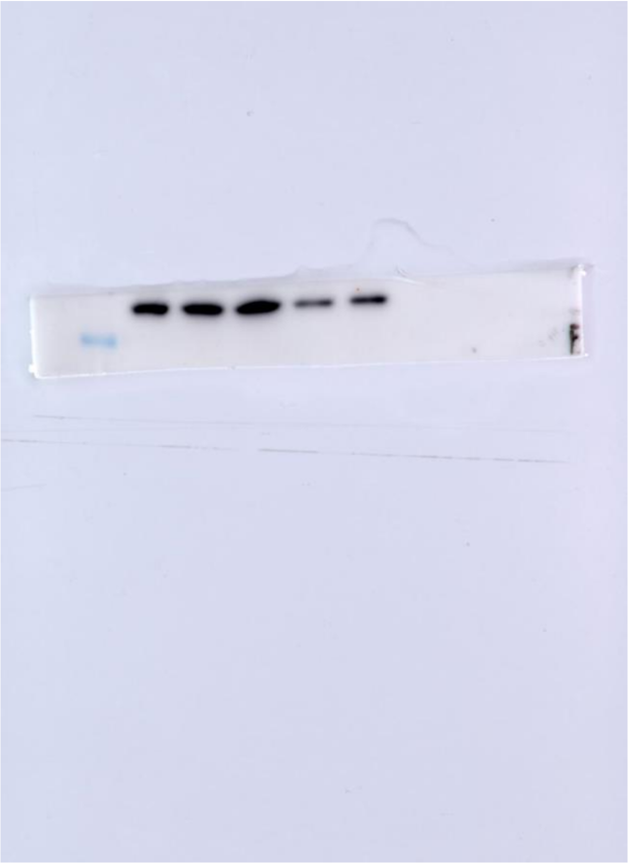

Second time

β-ACTIN

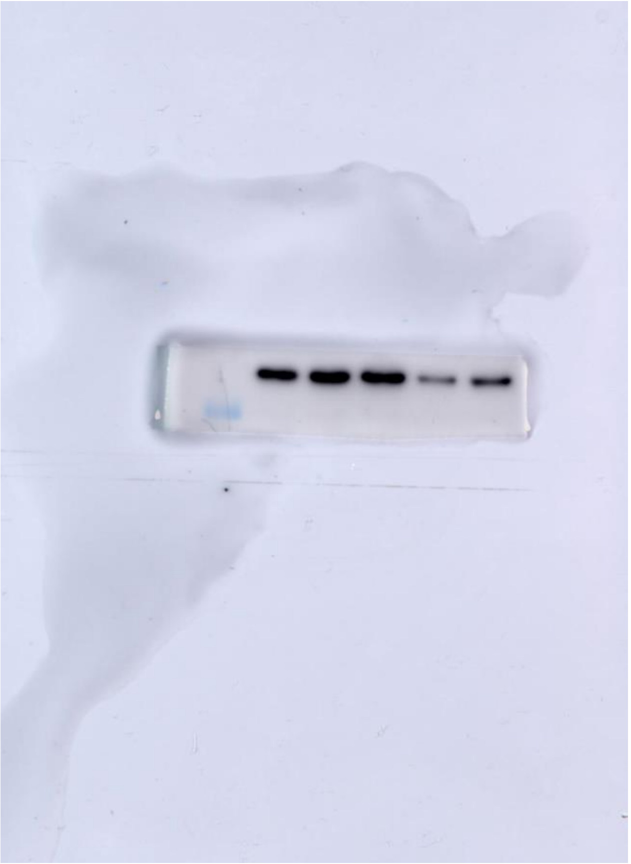

Akt

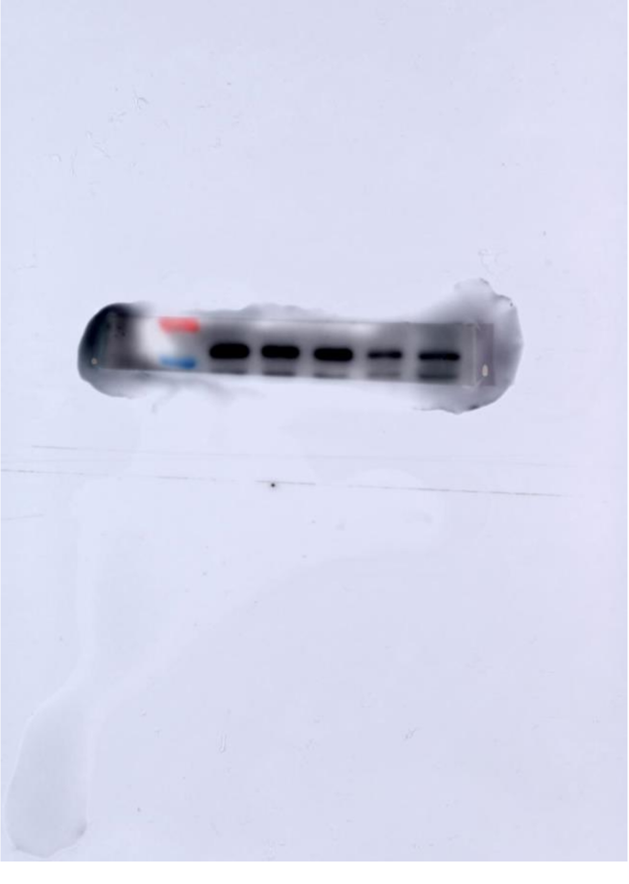

Pho-Akt

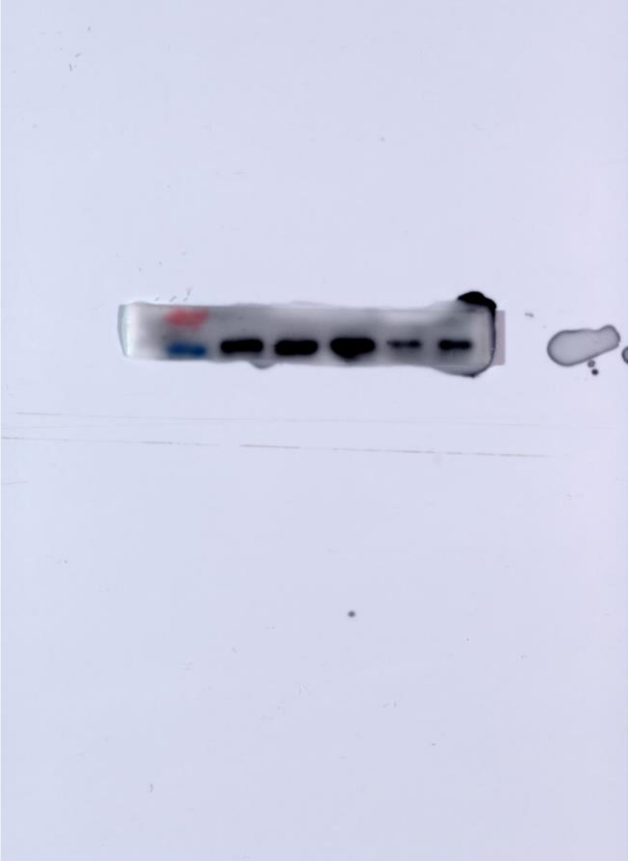

SRPK2

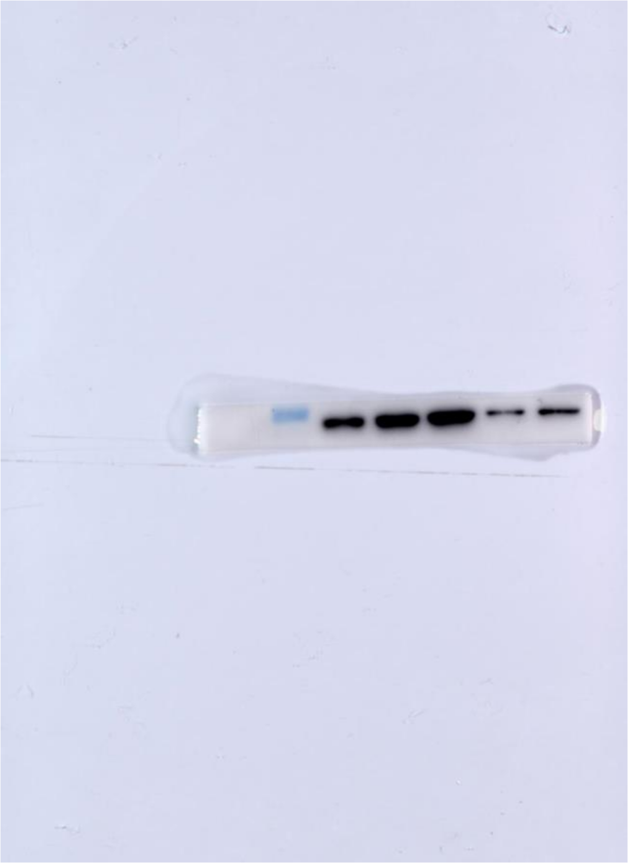

Pho-SRPK2

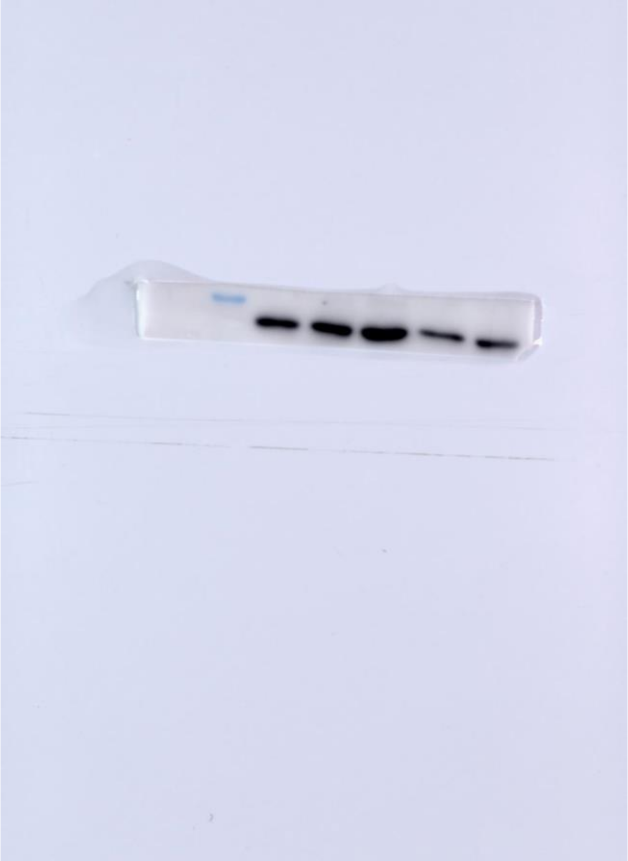

ACIN

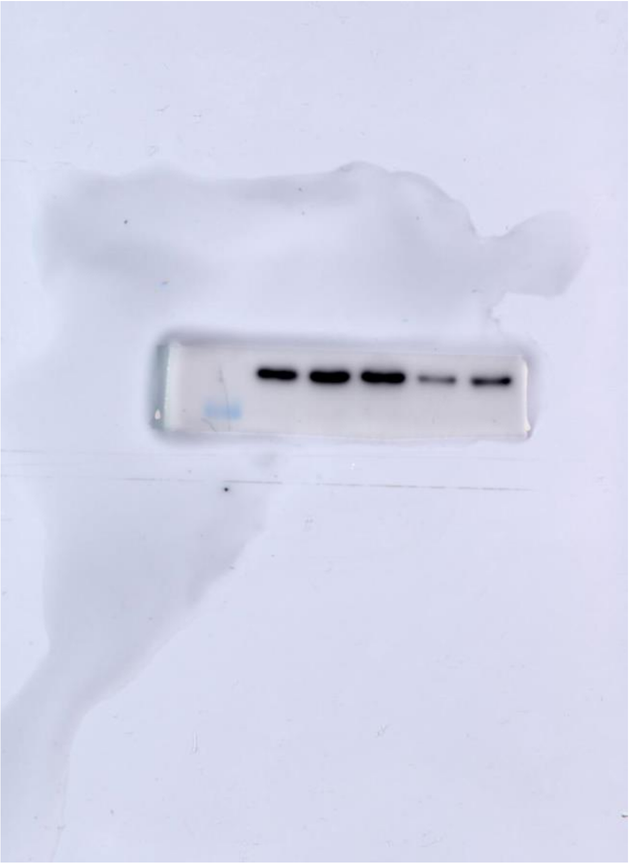

Third time

β-ACTIN

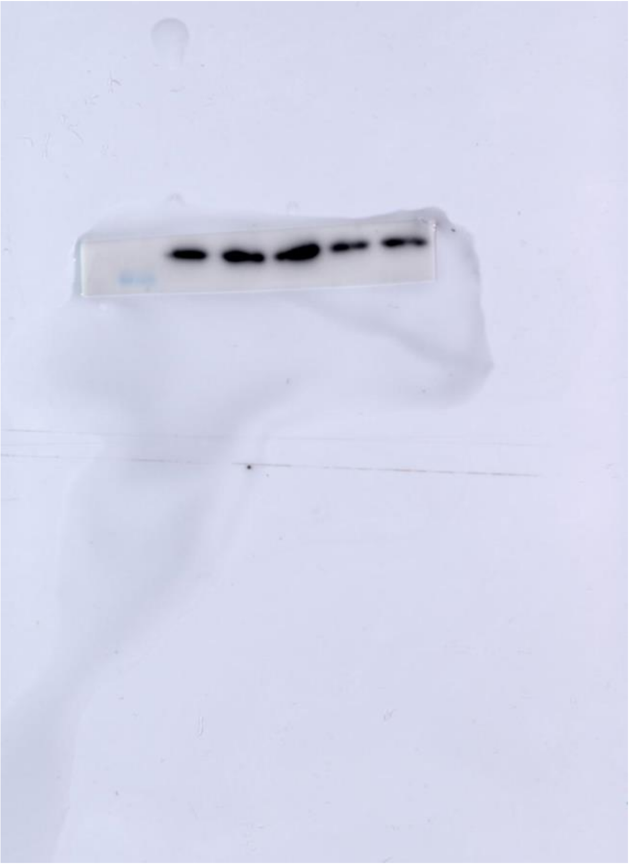

Akt

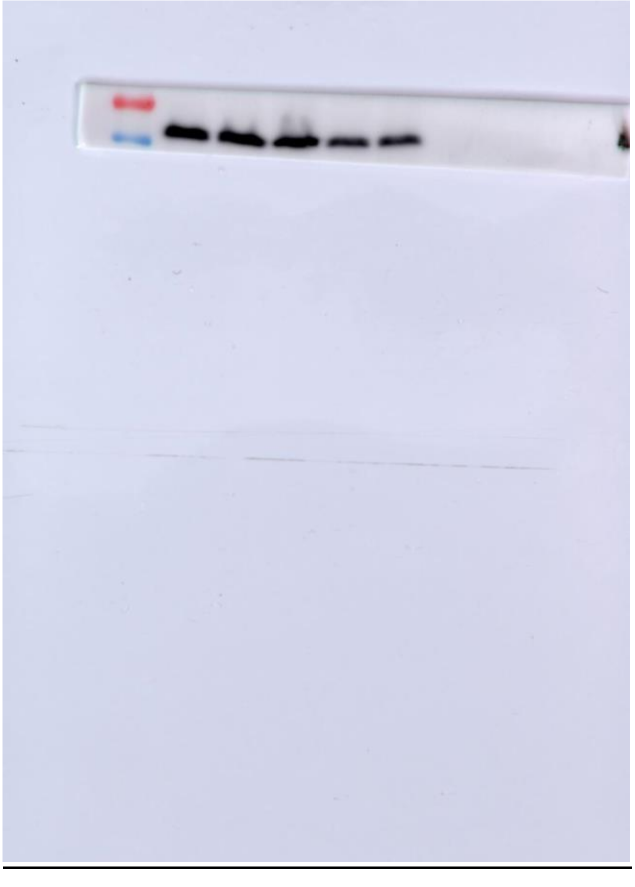

Pho-Akt

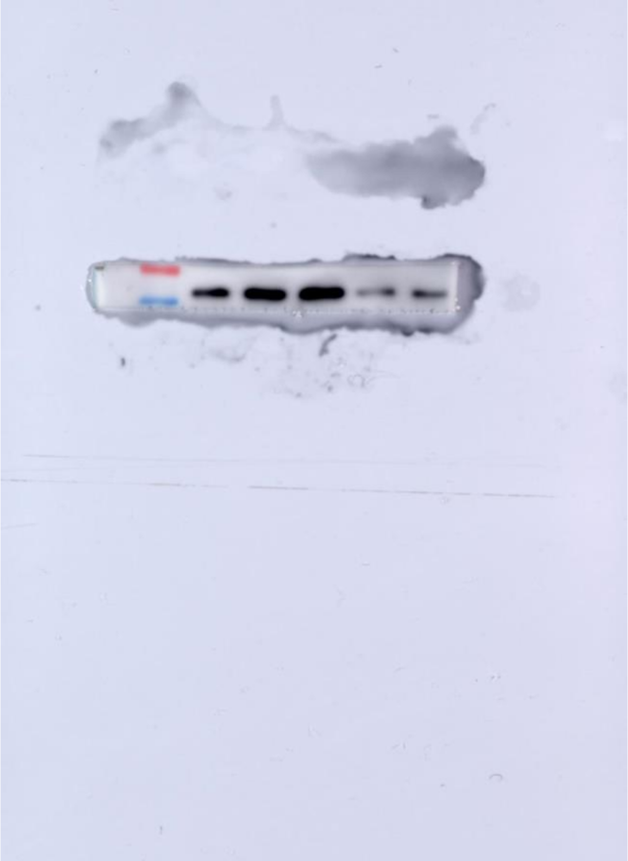

SRPK2

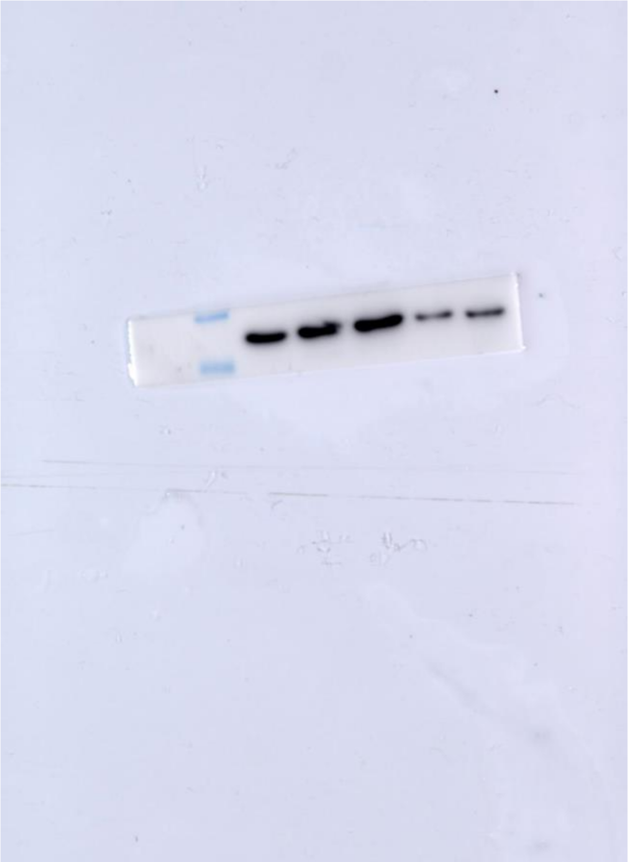

Pho-SRPK2

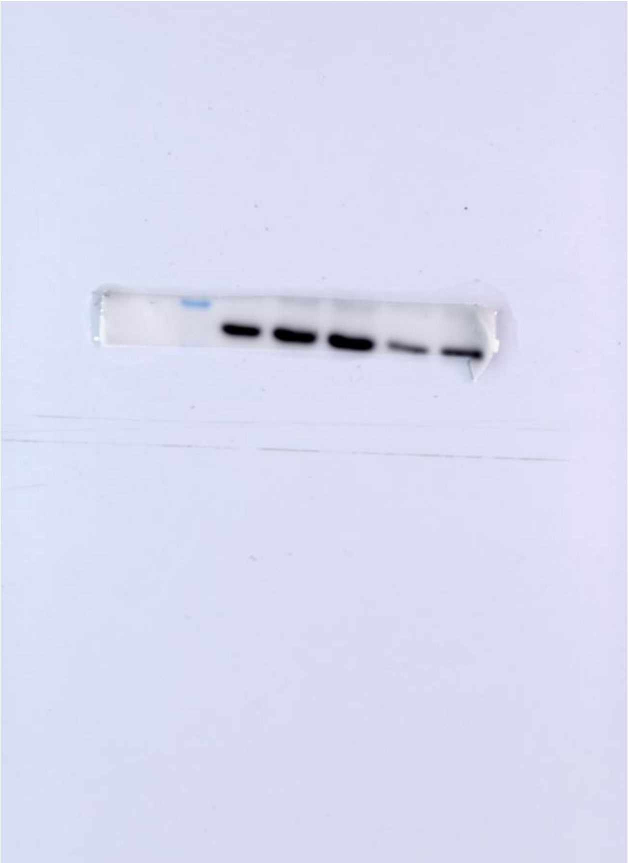

ACIN

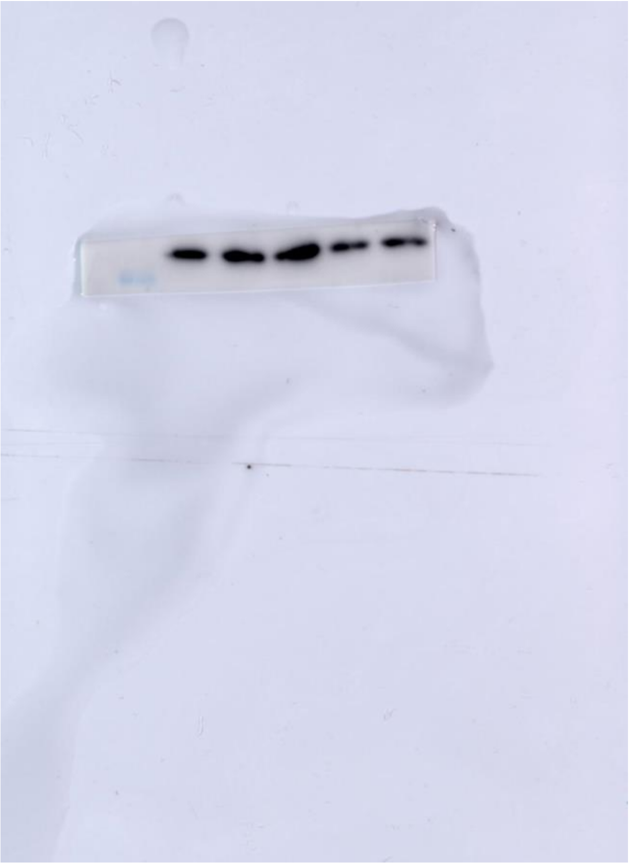

Supplement: Supplementary file 1 [file Image_1.pdf]
