# Supplementary figures and images for: SRPK2 Expression and Beta-Amyloid Accumulation Are Associated With BV2 Microglia Activation
Source: Front Integr Neurosci. 2022 Jan 28;15:742377. doi: 10.3389/fnint.2021.742377 (PMC8831369; doi:10.3389/fnint.2021.742377)

a

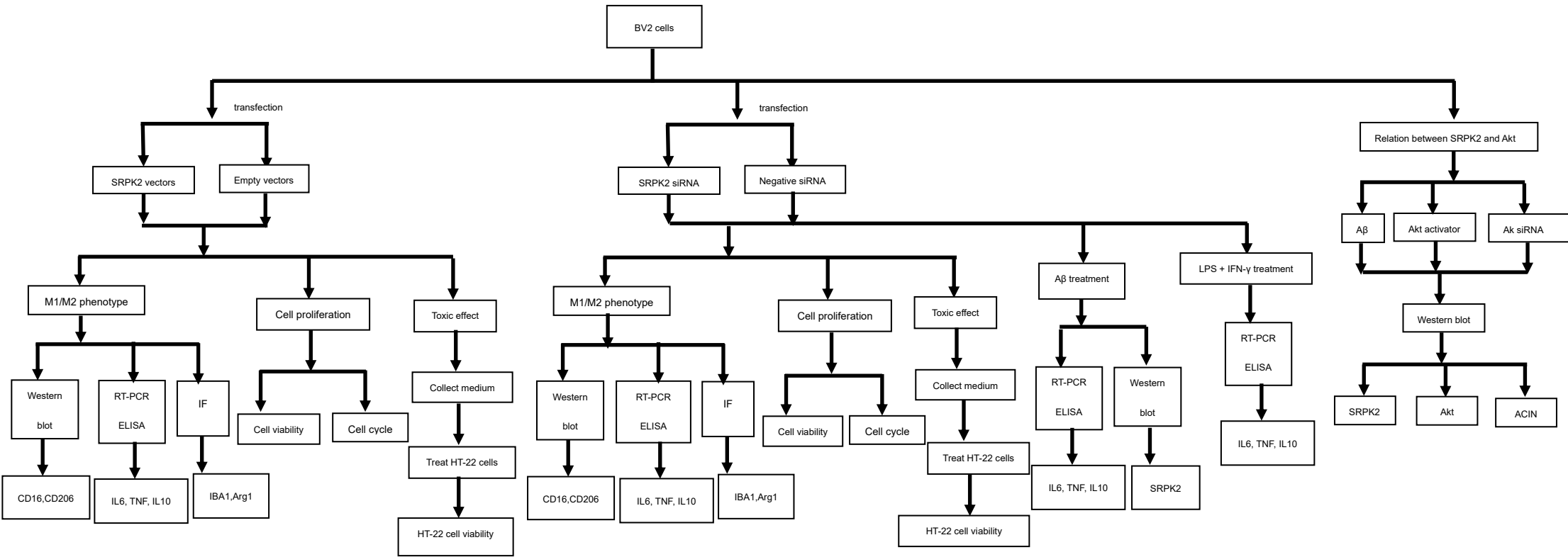

Supplement: Supplementary file 2 [file Data_Sheet_1.PDF]
