# Supplementary material for: SRPK2 Expression and Beta-Amyloid Accumulation Are Associated With BV2 Microglia Activation
Source: Front Integr Neurosci. 2022 Jan 28;15:742377. doi: 10.3389/fnint.2021.742377 (PMC8831369; doi:10.3389/fnint.2021.742377)
Supplement: Supplementary file 3 [file Table_1.pdf]

<table>
<tr><td colspan="4">Normality test data</td></tr>
<tr><td colspan="2"></td><td></td><td></td></tr>
<tr><td colspan="2"></td><td>Kolmogorov–Smirnov test</td><td>P value</td></tr>
<tr><td colspan="2">Figure 1A</td><td></td><td></td></tr>
<tr><td colspan="2">SRPK2-en VS. empty vector</td><td></td><td></td></tr>
<tr><td colspan="2">SPRK2</td><td>0.311</td><td>0.071</td></tr>
<tr><td colspan="2">Pho-SRPK2</td><td></td><td></td></tr>
<tr><td colspan="2">CD16/32</td><td>0.294</td><td>0.114</td></tr>
<tr><td colspan="2">Cd206</td><td>0.233</td><td>0.200</td></tr>
<tr><td colspan="2">Figure 1B</td><td></td><td></td></tr>
<tr><td colspan="2">SRPK2-siRNA VS. negative siRNA</td><td></td><td></td></tr>
<tr><td colspan="2">SPRK2</td><td>0.293</td><td>0.116</td></tr>
<tr><td colspan="2">Pho-SRPK2</td><td></td><td></td></tr>
<tr><td colspan="2">CD16/32</td><td>0.279</td><td>0.158</td></tr>
<tr><td colspan="2">Cd206</td><td>0.304</td><td>0.087</td></tr>
<tr><td colspan="2">SRPK2-en VS. empty vector</td><td></td><td></td></tr>
<tr><td colspan="2">(Figure 1C) IBA1</td><td>0.085</td><td>0.200</td></tr>
<tr><td colspan="2">(Figure 1D) Arg1</td><td>0.121</td><td>0.063</td></tr>
<tr><td colspan="2">(Figure 1E) %amoeboid</td><td>0.071</td><td>0.200</td></tr>
<tr><td colspan="2">SRPK2-siRNA VS. negative siRNA</td><td></td><td></td></tr>
<tr><td colspan="2">(Figure 1F) IBA1</td><td>0.047</td><td>0.200</td></tr>
<tr><td colspan="2">(Figure 1G) Arg1</td><td>0.038</td><td>0.200</td></tr>
<tr><td colspan="2">(Figure 1H) %amoeboid</td><td>0.068</td><td>0.200</td></tr>
<tr><td colspan="2">Figure 2</td><td></td><td></td></tr>
<tr><td rowspan="6">SRPK2-en VS. empty vector</td><td>IL-6 RNA level</td><td>0.240</td><td>0.310</td></tr>
<tr><td>TNF-α RNA level</td><td>0.316</td><td>0.062</td></tr>
<tr><td>IL-10 RNA level</td><td>0.297</td><td>0.106</td></tr>
<tr><td>IL-6 protein level</td><td>0.243</td><td>0.200</td></tr>
<tr><td>TNF-α protein level</td><td>0.277</td><td>0.166</td></tr>
<tr><td>IL-10 protein level</td><td>0.173</td><td>0.200</td></tr>
<tr><td rowspan="6">SRPK2-siRNA VS. negative siRNA</td><td>IL-6 RNA level</td><td>0.303</td><td>0.090</td></tr>
<tr><td>TNF-α RNA level</td><td>0.251</td><td>0.200</td></tr>
<tr><td>IL-10 RNA level</td><td>0.270</td><td>0.196</td></tr>
<tr><td>IL-6 protein level</td><td>0.160</td><td>0.200</td></tr>
<tr><td>TNF-αprotein level</td><td>0.291</td><td>0.124</td></tr>
<tr><td>IL-10 protein level</td><td>0.160</td><td>0.200</td></tr>
<tr><td colspan="2">Figure 3A</td><td></td><td></td></tr>
<tr><td colspan="2">SRPK2-en VS. empty vector</td><td>0.161</td><td>0.200</td></tr>
</table>

SRPK2-siRNA VS. negative siRNA		0.262	0.200
**Figure 3B**			
Control VS. Aβ		0.162	0.200
Control VS. Aβ+SRPK2 KD		0.166	0.200
**Figure 4A**			
SRPK2-en VS. empty vector		0.203	0.200
SRPK2-siRNA VS. negative siRNA		0.155	0.200
**Figure 4B**			
Control VS. Aβ		0.180	0.200
Control VS. Aβ+SRPK2 KD		0.229	0.189
**Figure 5**			
Control VS. Aβ	SRPK2	0.283	0.145
	Pho-SRPK2	0.260	0.200
**Figure 6A**			
Control VS. Aβ	IL-6 RNA level	0.305	0.085
	TNF-α RNA level	0.311	0.072
	IL-10 RNA level	0.284	0.141
	IL-6 protein level	0.311	0.071
	TNF-α protein level	0.276	0.172
	IL-10 protein level	0.217	0.200
Control VS. LPS	IL-6 RNA level	0.310	0.075
	TNF-α RNA level	0.307	0.081
	IL-10 RNA level	0.307	0.081
	IL-6 protein level	0.297	0.105
	TNF-α protein level	0.299	0.100
	IL-10 protein level	0.187	0.200
**Figure 6B**			
Negative siRNA VS. LPS+Negative siRNA VS. LPS+SPRK2 KD		0.118	0.200
Negative siRNA VS.Aβ+Negative siRNA VS. Aβ+SPRK2 KD		0.143	0.200
**Figure 7**			
Akt		0.131	0.200
SRPK2		0.153	0.200
ACIN1		0.119	0.200
Pho-Akt		0.127	0.200
Pho-SRPK2		0.160	0.200
